# In-depth population genetic study of *Vitis vinifera* ssp. *sylvestris* from the Black Sea region and its virome

**DOI:** 10.3389/fpls.2025.1536862

**Published:** 2025-03-25

**Authors:** Daria Belkina, Ilya Stepanov, Marina Makarkina, Elena Porotikova, Ilya Lifanov, Evgeniy Kozhevnikov, Svetlana Gorislavets, Svetlana Vinogradova

**Affiliations:** ^1^ Skryabin Institute of Bioengineering, Research Center of Biotechnology of the Russian Academy of Sciences, Moscow, Russia; ^2^ North Caucasian Federal Scientific Center of Horticulture, Viticulture, Wine-Making, Krasnodar, Russia; ^3^ Grape Genome Research Laboratory, All-Russian National Research Institute of Viticulture and Winemaking “Magarach” Russian Academy of Sciences (RAS), Yalta, Russia

**Keywords:** phylogeography, virome, HTS, metagenomics, novel virus, wild grapevine, grapevine viruses, *Vitis vinifera* ssp. *sylvestris*

## Abstract

The spread of cultivated grapevine from primary centers of origin is inevitably accompanied by the range expansion of its pathogens, including viruses. A limited number of wild *Vitis vinifera* L. ssp. *sylvestris* (Gmelin) Hegi populations have survived in the centers of grapevine domestication and can be used for comprehensive studies. We analyzed 50 grapevines collected in protected areas of the Black Sea region, which belong to the Caucasian domestication center. Based on genotyping of grapevines using simple sequence repeats as DNA markers, we determined the phylogenetic placement of *V. vinifera* ssp. *sylvestris* from the Black Sea region compared to cultivated and wild grapevines of the world. Using high-throughput sequencing of total RNA, we obtained the viromes of these grapevines. Ten viruses and one viroid were identified. The most common viruses detected were Vitis cryptic virus, grapevine rupestris stem pitting–associated virus, grapevine Pinot gris virus, and grapevine virus T. Among the economically significant viruses, we identified grapevine leafroll-associated virus 1 and grapevine virus A. A total of 91 complete or nearly complete virus genomes and one viroid genome were assembled, and phylogenetic analysis was performed. Two novel (+) ssRNA viruses were discovered, tentatively named Abrau grapevine-associated virus in the order *Hepelivirales* and Taurida grapevine-associated virus in the order *Picornavirales*. It is important to comprehensively consider the phylogeography of both viruses and their plant hosts. This is the first study that simultaneously addresses the population genetics of *V. vinifera* ssp. *sylvestris* from the Caucasian domestication center and its viruses.

## 1 Introduction

Grapevine (*Vitis vinifera* L.) is one of the most important agricultural crops and is closely associated with the cultural heritage of humankind. *V. vinifera* has two subspecies: *V. vinifera*. ssp. *vinifera* (or *sativa*) (DC.) Hegi (cultivated grape) and *V. vinifera* ssp. *sylvestris* (Gmel.) Hegi (wild grape). The ancestor of cultivated grapevine is *V. vinifera* ssp. *sylvestris* (Magris et al., 2021), which now grows in forested areas from the Atlantic coast of Western Europe and North Africa to the Himalayas (This et al., 2006).

Studying the process of grapevine domestication is difficult due to the small number of surviving populations of *V. vinifera* ssp. *sylvestris* (Arnold et al., 1998). The most valuable are the populations that have survived to this day in the centers of domestication: the Mediterranean (Arroyo-García et al., 2006), the Caucasus (McGovern et al., 2017; Riaz et al., 2018), and Central Asia (Riaz et al., 2018). Currently, the populations of *V. vinifera* ssp. *sylvestris* in Europe are endangered because of the spread of pathogens as a result of human activity and increased forest fires (Butorac et al., 2018). The rapid reduction of its range is accompanied by a decrease in genetic diversity (Zdunić et al., 2017) and erosion of the gene pool due to gene flow from cultivated varieties (Di-Vecchi-Staraz et al., 2009; Lopes et al., 2009; Meléndez et al., 2016). One of the few regions where natural populations of *V. vinifera* ssp. *sylvestris* have survived in the Black Sea region. This region overlaps with the Caucasian center of grapevine domestication. Previously, local populations of wild grapevine were studied from a morphological and genetic perspective (Gorislavets et al., 2021; Ilnitskaya et al., 2023).

Currently, active work is underway to monitor, study, and preserve *V. vinifera* ssp. *sylvestris* as a potential source of economically valuable traits for the selection of cultivars resistant to abiotic and biotic factors (Šiško et al., 2021; Perko et al., 2024). In addition, these ecosystems may harbor unknown species of organisms, including viruses. Several years ago, grapevine foveavirus A (*Foveavirus alphavitis*) was discovered in *V. vinifera* ssp. *sylvestris* (Reynard et al., 2020). Other *Vitis* species were found to be the host of wild Vitis virus 1 (*Grablovirus silvestris*) (Perry et al., 2018), Vitis varicosavirus (*Varicosavirus vitis*), Vitis emaravirus (*Emaravirus vitis*), and Vitis cryptic virus (VCV) (Nabeshima and Abe, 2021). These viruses and others yet to be discovered may be potential pathogens of grapevine diseases. However, at the current stage of scientific development, it is clear that the relationship between viruses and the host plant is not always strictly parasitic but can be mutualistic (Roossinck and Bazán, 2017). As a rule, viruses exert a positive effect only under certain environmental conditions, which is called conditional mutualism (Bronstein, 1994; Hily et al., 2016). For example, viruses are capable of increasing the resistance of the host plant to abiotic stresses, such as drought (Xu et al., 2008). In addition, the impact of certain viruses on the production of volatile compounds by plants modulates their attractiveness to herbivorous insects (van Molken et al., 2012; Shapiro et al., 2013; Bera et al., 2020). These effects can be mediated by specific viral proteins that influence phytohormone production (Aguilar and Lozano-Duran, 2022; Prakash et al., 2023). The virome of *V. vinifera* ssp. *sylvestris* may contain previously undescribed mutualistic viruses that have co-evolved with wild grapevines for centuries.

Most of the studies of wild grapevine viruses available to date have focused on the search for specific pathogens. As a rule, economically significant viruses are rarely found in wild grapevine; therefore, wild populations are unlikely to pose a major threat to viticulture. In the Mediterranean region, grapevine viruses such as grapevine fanleaf virus (GFLV; *Nepovirus foliumflabelli*), Grapevine leafroll-associated virus 1 (GLRaV-1; *Ampelovirus univitis*), grapevine leafroll-associated virus 3 (GLRaV-3; *Ampelovirus trivitis*), grapevine virus A (GVA; *Vitivirus alphavitis*), and grapevine rupestris stem pitting–associated virus (GRSPaV; *Foveavirus rupestris*) have been occasionally detected in local populations of *V. vinifera* ssp. *sylvestris* using polymerase chain reaction (PCR) and ELISA (Pacifico et al., 2016; Sabella et al., 2018; Selmi et al., 2020), whereas GLRaV-1 has been detected in a population localized along the Danube (Regner et al., 2004). More systematic studies involving sRNA and total RNA sequencing are being conducted on North American *Vitis* species (Hu et al., 2021; Thompson et al., 2021).

Modern methods based on the use of high-throughput sequencing can provide data on the phylogeography of viruses that will allow a new look at the routes of grapevine dissemination from domestication centers. We report the first comprehensive study of a population of *V. vinifera* ssp. *sylvestris* from the Caucasian center of grapevine domestication; the composition, diversity, and phylogeny of viruses that accompany wild grapevines in their natural habitat; and the population genetics of these grapevines and viruses.

## 2 Materials and methods

### 2.1 Sample collection

Samples of 50 wild grapevines were collected in three protected areas of the Black Sea region (**Supplementary Figure S1**). There were no symptoms of viral disease. In the Crimean Nature Reserve located on the southern coast of the Crimean Peninsula, 18 grapevine samples were collected at two locations near the cities of Alushta and Yalta. In the Utrish State Nature Reserve located on the Abrau Peninsula and adjacent territories, 27 samples were collected at four locations: Lobanova Shchel, Vodopadnaya Shchel, Shirokaya Shchel, and Shkolny Khutor. The samples were transported to the laboratory at +4°C to 8°C and then stored at −20°C. An additional five samples were collected from the mountain forests of Abkhazia. These samples were stored in dried form. Detailed sample information is provided in **Supplementary Table S1**.

### 2.2 SSR genotyping of wild grapevine

Plant DNA was extracted from the vine and leaves using the CTAB method (Rogers and Bendich, 1985). DNA quality was verified using a Nano-500 spectrophotometer (Allsheng, Hangzhou, China).

Genotyping was performed at nine microsatellite markers: VVS2, VVMD5, VVMD7, VVMD25, VVMD27, VVMD28, VVMD32, VrZAG62, and VrZAG79, as recommended by the OIV for DNA profiling of grapevine (This et al., 2004). Pinot noir and Chardonnay were used as reference cultivars of *V. vinifera*, the DNA profiles of which at nine loci are available in the international database Vitis International Variety Catalogue (VIVC). Flower sex was determined using the VVIb23 marker associated with the sex locus (Merdinoglu et al., 2005). PCR products 284 bp in size were considered to correspond to the hermaphroditism allele (H), 288 bp corresponded to the female allele (F), and 304 bp corresponded to the male allele (M) (Battilana et al., 2013). Kishmish Vatkana was used as a reference cultivar for the VVIb23 marker. The primers used for the analysis are listed in **Supplementary Table S2**. PCR was carried out using reagents from SibEnzyme (Moscow, Russia). The 20 µl reaction mixture included 1× Taq polymerase buffer, 0.1% BSA, 0.4 mM each dNTP, 1 U Taq polymerase, 0.3 µM each primer, and 10–40 ng of DNA template. PCR was carried out using a MasterCycler Nexus GX2 (Eppendorf, Hamburg, Germany). The amplification products were separated via capillary electrophoresis using a Nanofor 05 Genetic Analyzer and built-in software (Institute of Analytical Instrumentation of RAS, St. Petersburg, Russia). The sizes of the target alleles of the SSR loci were determined via comparison with the molecular weight marker CD-600 (Syntol, Moscow, Russia).

### 2.3 Population genetic analysis of wild grapevine

A population genetic analysis of samples of wild grapevine from the Black Sea region was carried out using DNA profiles at nine SSR loci. The analysis also included 61 SSR profiles of previously genotyped samples from the Crimean and Utrish nature reserves (**Supplementary Table S3**) (Ilnitskaya et al., 2023, 2024; Korniliev, 2023), 119 SSR profiles of cultivars from the VIVC database, and 540 SSR profiles of *V. vinifera* ssp. *sylvestris* from previous studies (**Supplementary Tables S4** and **S5**) (De Andrés et al., 2012; Riaz et al., 2018; D’Onofrio, 2020; Zdunić et al., 2020; Rahimi et al., 2021; Perko et al., 2024). All of them were normalized by SSR profiles of the Cabernet Sauvignon, Pinot Noir, Syrah, Chardonnay Blanc, and Riesling Weiss varieties. To obtain more even sampling, the final dataset was formed in such a way that it covered a comparable number of samples from each population and characterized its genetic diversity.

A statistical analysis of data on polymorphisms of microsatellite loci was carried out for the groups of grapevine using the average values of the number of alleles per locus (Na), the effective number of alleles per locus (Ne), and the Shannon diversity index (I). The parameters of expected heterozygosity (He), observed heterozygosity (Ho), and Wright’s fixation index (F) were calculated using the Genalex v. 6.5 software (Peakall and Smouse, 2012).

The degree of genetic differentiation (Fst) and level of gene flow (Nm) between wild grapevine populations from the Black Sea region and other groups of grapevine were calculated using Genalex v. 6.5 software. Principal Coordinates Analysis (PCoA) was performed with PAST v. 4.17 software using the Dice similarity index (Hammer et al., 2001). The PCoA projections were represented in a two-dimensional scatter plot. Genetic relatedness between grapevine groups was assessed by the UPGMA method using POPGENE 1.32 software (Yeh and Boyle, 1997).

A Bayesian analysis was performed using STRUCTURE 2.3.4 software with the following settings: burn-in period = 100,000, reps = 100,000, and number of iterations = 5 (Pritchard et al., 2000). The optimal number of clusters was calculated with Structure Harvester software using deltaK values, as defined in Evanno et al. (2005).

### 2.4 Total RNA sequencing

Total RNA was extracted from grapevine leaves and petioles using CTAB buffer (Morante-Carriel et al., 2014). Total RNA extraction was conducted on individual plants. The quality of the RNA was verified using an Eppendorf BioSpectrometer (Eppendorf, Hamburg, Germany) and electrophoresis in a 1.2% agarose gel. The RNA concentration was measured on a Qubit 3.0 fluorometer (Invitrogen, Waltham, MA, USA) using the Qubit RNA BR Assay Kit (Thermo Fisher Scientific, Waltham, MA, USA).

Prior to library preparation, total RNA was subjected to DNase I treatment (Thermo Fisher Scientific, Waltham, MA, USA), and plant ribosomal RNA was depleted using the RiboMinus Plant Kit (Thermo Fisher Scientific, Waltham, MA, USA).

cDNA sequencing libraries were prepared using the QIAseq Stranded RNA Library Kit (Qiagen, Hilden, Germany) and NEBNext Ultra II DNA Library Prep Kit for Illumina (New England Biolabs, Ipswich, MA, USA). The DNA concentration was measured using a Qubit dsDNA HS Assay Kit (Thermo Fisher Scientific, Waltham, MA, USA) on the Qubit 3.0 fluorometer (Invitrogen, Waltham, MA, USA). The quality of the libraries was assessed using a Bioanalyzer 2100 (Agilent Technologies, Santa Clara, CA, USA). The prepared libraries were paired-end sequenced (2 × 150 bp) on a NovaSeq 6000 System (Illumina, San Diego, CA, USA). The bioproject containing the sequenced libraries was submitted to GenBank under accession number PRJNA1148509.

### 2.5 Bioinformatics analysis of sequencing data

Bioinformatics processing of the HTS data was performed using Geneious Prime v. 2023.2.1 software (Biomatters, Ltd., Auckland, New Zealand). Low-quality and adapter sequences were removed using the BBDuk plugin (minQ = 30, min length = 10 bp). The trimmed reads were merged, and duplicates were removed at kmer = 30.

Taxonomic classification of the preprocessed reads was performed using the Kraken2 tool (Wood et al., 2019) as described by Haegeman et al. (2023). The PlusPFP database (12/1/2024) with a size of 171 GB containing reference genomes of archaea, bacteria, viruses, plasmids, protozoa, fungi, plants, and humans was used (Index zone by Ben Langmead, 7 October 2024).

Preprocessed reads were mapped to reference genomes of grapevine viruses and viroids (latest update of the genome list: March 2024) with medium-low sensitivity (maximum mismatches per read = 20%, word length = 18). To prevent false-positive identification, a threshold of 10 reads with 15% reference coverage was set for viruses with a large number of reads: GRSPaV, grapevine pinot gris virus (GPGV; *Trichovirus pinovitis*), and grapevine virus T (GVT; *Foveavirus tafvitis*).

*De-novo* assembly was performed using two assemblers, SPAdes, and Geneious, with default settings. Contigs were compared to the NCBI reference viral genome database (accessed on 15 September 2022) using the TBLASTX algorithm. In further analysis, only contigs with *E*-value ≤ 1 × 10^−40^ were considered.

Consensus sequences obtained after mapping reads to reference genomes or whole-genome contigs (if they were available) were used to assemble the genomes of viruses and viroid. If there were gaps in the consensus sequence, we performed a BLASTN search against the GenBank database and selected the closest genome. This genome was used as a reference for remapping. The consensus sequences contained only sample-specific data. Genomes were considered assembled when they covered more than 90% of the reference sequence and had ambiguities of less than 5% of the consensus length. The GenBank accession numbers of the assembled genomes are provided in **Supplementary Table S6**.

The results of the bioinformatics analysis were visualized using the ggplot2 and ggnet2 packages in the R programming language in the RStudio integrated development environment.

### 2.6 Discovery and analysis of novel grapevine-associated viruses

Plant virus contigs that showed matches with unexpected grapevine viruses after TBLASTX analysis were analyzed to search for novel grapevine viruses. These contigs were searched against the GenBank database using BLASTN and BLASTX. For further analysis, we used contigs that did not give any matches to sequences from the database using BLASTN but were similar to viral sequences using BLASTX. These contigs were used to map reads from each library; upon successful mapping, all preprocessed reads from these libraries were combined into one data pool. The reads that mapped to the *V. vinifera* genome (GCF_000003745) were removed from this pool, followed by *de-novo* assembly using SPAdes and Geneious assemblers. The resulting contigs were analyzed using TBLASTX against the NCBI reference viral genome database as well as a local database containing nucleotide sequences of viruses of the family or the order to which the previously identified contigs belong.

All the selected contigs were extended by mapping reads from all the libraries onto them with several iterations and aligned to the genome of the closest virus. Gaps between contigs were filled by bidirectional Sanger sequencing using the BigDyeTM Terminator v3.1 Cycle Sequencing Kit (Thermo Fisher Scientific, Waltham, MA, USA) on an ABI PRISM 3730 automated sequencer (Applied Biosystems, Foster, CA, USA). The primers are listed in **Supplementary Table S2**. The sequences were analyzed using Finch TV 1.4.0 (Geospiza Inc., Seattle, WA, USA).

The 5’ and 3’ ends of the novel virus were identified using the Step-Out rapid amplification of cDNA ends (Step-Out RACE) (Matz, 1999); cDNAs were synthesized using the Mint cDNA Synthesis Kit (Cat. #SK001, Evrogen, Moscow, Russia) and total RNA as a template. Step-by-step cDNA amplification was then performed using the external priming technique with universal adapter primers from the Mint RACE primer set (Cat. #SK004, Evrogen, Moscow, Russia) and 5’ and 3’ ends specific primers (**Supplementary Table S2**). The primers were checked for secondary structures using the Beacon Designer free tool (Premier Biosoft International, San Francisco, CA, USA). The polymerase from the Encyclo Plus PCR Kit was used for amplification (Cat. #PK001, Evrogen, Moscow, Russia). The amplification products were separated by electrophoresis in a 1.2% agarose gel, excised using the Cleanup Standard Kit (Evrogen, Moscow, Russia), and cloned into the pAL2-T vector using the Quick-TA Kit (Cat. #TAK02, Evrogen, Moscow, Russia). After chemical transformation of competent *Escherichia coli* XL1-Blue cells (Cat. #CC001, Evrogen, Moscow, Russia), the cells were cultured on LB medium supplemented with ampicillin, XGal, and IPTG (Casali et al., 2003). Cloned DNA fragments were sequenced using the primers M13F and M13R from the Quick-TA Kit.

The full genome sequence of a novel virus was assembled based on the nucleotide sequences from the same library to eliminate polymorphisms of different isolates from the sequence. The assembled genomes were deposited in GenBank (**Supplementary Table S6**).

The genome annotation of novel viruses was carried out using the NCBI ORFfinder tool. The prediction of conserved protein domains was performed using the NCBI Conserved Domain Search and InterPro database (Paysan-Lafosse et al., 2023). A pairwise comparison of the sequences of novel viruses with the closest known viruses was performed using the Clustal W alignment algorithm in the Sequence Demarcation Tool (SDT) (Muhire et al., 2014).

### 2.7 Validation of grapevine viruses and viroid

The presence of each bioinformatically identified virus and viroid was verified in all samples using RT-PCR. cDNAs were synthesized using 1 μg of total RNA as a template, random hexamers, and RevertAid H Minus Reverse Transcriptase (Thermo Fisher Scientific, Waltham, MA, USA). As a control for successful cDNA synthesis, a fragment of the 18S rRNA gene was amplified. The reaction mixture for RT-PCR comprised 0.2 mM of each dNTP, 1× Taq Buffer with (NH_4_)_2_SO_4_, 2.5 mM MgCl2, 1 µM of each primer, and 0.375 U of Taq polymerase (Thermo Fisher Scientific, Waltham, MA, USA). All primers are listed in **Supplementary Table S2**. The PCR products were visualized in a 1.2% agarose gel with the 100+ bp DNA Ladder (Evrogen, Moscow, Russia).

In cases where bioinformatics identification coincided with PCR validation, the sample was considered virus-positive.

### 2.8 Phylogenetic, recombination, and population genetic analysis of viruses

Nucleotide sequences for phylogenetic analysis of the viruses identified in this study were downloaded from GenBank. Multiple sequence alignments were performed using the Clustal Omega 1.2.2 algorithm. The optimal model of DNA sequence evolution was determined in MEGA11 software (Tamura et al., 2021). Dendrograms were constructed in MEGA11 using the maximum likelihood method with 1,000 bootstrap replicates. The tree was rooted with an outgroup that was selected individually for each virus among related taxa. Detailed information about the settings for each dendrogram is provided in **Supplementary Table S7**.

Population genetic parameters were estimated for the most widely represented viruses in the studied samples (VCV, GPGV, GVT, and GRSPaV). For the analysis of VCV, GPGV, and GVT, we used sequences prepared for phylogenetic analysis. Representatives of small populations and isolates with unknown countries of origin were removed. For GRSPaV analysis, we used >95% assembled genomes from GenBank. Sequences aligned by the Clustal Omega algorithm were cut off at both ends.

Probable recombination events were determined in RDP v. 4.101 software (Martin et al., 2015) using RDP, GENECONV, Chimaera, MaxChi, BootScan, SiScan, and 3Seq algorithms. In further analysis, only recombination events confirmed by five or more algorithms were considered.

The assessment of nucleotide diversity in populations was performed using MEGA11. The diversity index π was calculated as the average number of nucleotide differences per site between any two sequences randomly selected from the population (Nei, 1987). To test the hypothesis of whether populations are affected only by neutral evolution or they are under selection pressure, we performed Tajima’s D analysis (Tajima, 1989) in Arlequin v. 3.5.2.2 software (Excoffier and Lischer, 2010). At D > 0, the population was considered to be under balancing selection, which maintains genetic polymorphism; at D < 0, the population was assumed to have recently passed through a bottleneck or was under purifying selection; at D = 0, the population was considered to evolve under the influence of random processes in accordance with neutral evolution.

Analysis of molecular variance and calculation of the proportion of genetic diversity due to differences among populations (Φst) were performed in Arlequin v. 3.5.2.2 software with the «Compute distance matrix» setting. The Φst index is an analog of Fst for haplotype data and can have values from 0 to 1 (Holsinger and Weir, 2009). At Φst → 0, populations were considered to be connected by frequent gene flow; at Φst → 1, the populations were considered to be genetically separated.

## 3 Results

### 3.1 Population genetic analysis of wild grapevine

An analysis of wild grapevine samples from the Black Sea region collected in this study using nine microsatellite markers revealed that the total number of identified alleles ranged from 8 (for VVMD25, VVMD27) to 13 (VVMD28), with an average of 10 alleles per marker (**Supplementary Table S8**). An analysis using the VVIb23 marker revealed that 37 samples carried the F allele (female), and 27 carried the M allele (male). The H allele (hermaphrodite) was found in one sample from the Utrish Nature Reserve (**Supplementary Table S9**). Another six alleles of different lengths, for which a statistically significant association with flower sex had not previously been established (Battilana et al., 2013), were detected in 20 samples. As a result, dioecy was reliably determined for 30 plants.

The highest genetic diversity was established for the wild grapevine population from the Utrish Nature Reserve: Na = 8.333, Ne = 4.211, whereas for the population from the Crimean Nature Reserve, these parameters were 6.000 and 3.191, respectively (**Supplementary Table S8**). The population from Abkhazia was the least diverse in terms of allele composition (Na = 3.333, Ne = 2.765). This is probably due to the uneven distribution of this population and the limited number of samples analyzed. The fixation index F for the three analyzed populations had negative values, indicating a high degree of heterozygosity.

**Figure 1:**
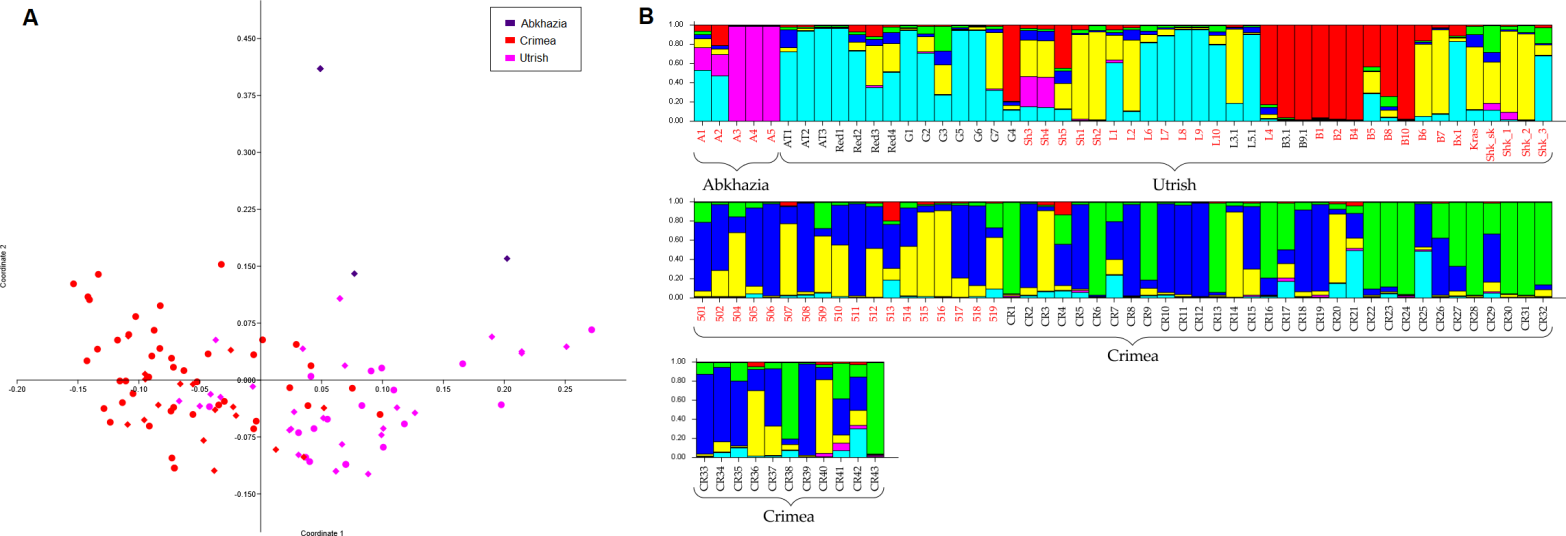
Population genetic analysis of wild grapevine from the Black Sea region using **(A)** the PCoA method and **(B)** STRUCTURE software at K = 6. Samples from this study are indicated by diamonds, and samples from previous studies are indicated by circles.

An analysis of the genetic kinship of the wild grapevine genotypes from the Black Sea region collected in this study and genotyped previously (**Supplementary Table S3**) by PCoA revealed that the population of grapevine from the Utrish Nature Reserve was the most genetically heterogeneous (**Figure 1A**). The distribution areas of samples from Crimean and Utrish nature reserves on the graph partially overlap, which may indicate the kinship of these populations.

Bayesian analysis of the population structure of wild grapevine from the Black Sea region generally revealed similar results. According to the data of the Structure Harvester, the population structure in this sample selection was best described at three and six clusters (**Supplementary Table S10**). At K = 3, the samples from Abkhazia grouped together with some of the samples from Utrish, and the other two clusters generally corresponded to the geographical division into the Utrish and Crimean populations (**Supplementary Figure S2**). At K = 6, the populations from Utrish and Crimea divided into three clusters each, and one of the clusters consisted of both Crimean and Utrish samples, which may indicate their genetic relationship (**Figure 1B**).

The populations of wild grapevine from the Black Sea region were used for pairwise comparisons of the Fst and Nm indices with the cultivars of *V. vinifera* ssp. *vinifera* and *V. vinifera* ssp. *sylvestris* from different geographic regions (**Supplementary Table S5**). Populations from the Crimean and Utrish nature reserves were genetically most similar to each other (Fst = 0.050) and were characterized by a high value of gene flow (Nm = 4.785). The closest to them in values of Fst and Nm were populations of wild grapevine from Italy and the Balkan Peninsula countries, along with a group of European cultivars (**Table 1**). At the same time, native varieties and wild grapevine from Crimea were genetically isolated from each other (Fst = 0.201, Nm = 0.992). Using the present dataset, the population of Abkhazian wild grapevine was genetically close to Abkhazian native varieties (Fst = 0.049, Nm = 4.897) and isolated from other populations of wild grapevine from the Black Sea region (Fst = 0.200–0.206, Nm = 0.967–0.997).

**Table 1:** Measurements of the genetic differentiation of wild grapevine populations from the Black Sea region.

Population	Wild grapevine Crimea		Wild grapevine Utrish		Wild grapevine Abkhazia	
	Fst	Nm	Fst	Nm	Fst	Nm
Wild grapevine Crimea	–	–	0.050	4.785	0.206	0.967
Wild grapevine Utrish	0.050	4.785	–	–	0.200	0.997
Wild grapevine Abkhazia	0.206	0.967	0.200	0.997	–	–
Abkhazian native varieties	0.144	1.485	0.126	1.734	0.049	4.897
Crimean native varieties	0.201	0.992	0.166	1.257	0.194	1.039
European varieties	0.096	2.354	0.085	2.697	0.123	1.778
Asian varieties	0.148	1.441	0.136	1.592	0.126	1.726
Wild grapevine Georgia	0.272	0.668	0.259	0.716	0.279	0.646
Wild grapevine Armenia	0.284	0.632	0.283	0.634	0.364	0.437
Wild grapevine Azerbaijan	0.290	0.613	0.283	0.632	0.319	0.533
Wild grapevine Israel	0.235	0.813	0.237	0.805	0.219	0.892
Wild grapevine Spain	0.177	1.163	0.188	1.083	0.210	0.939
Wild grapevine Italy	0.071	3.263	0.064	3.686	0.198	1.010
Wild grapevine Germany	0.228	0.849	0.185	1.101	0.455	0.299
Wild grapevine Balkans	0.067	3.502	0.053	4.444	0.213	0.924

**Figure 2:**
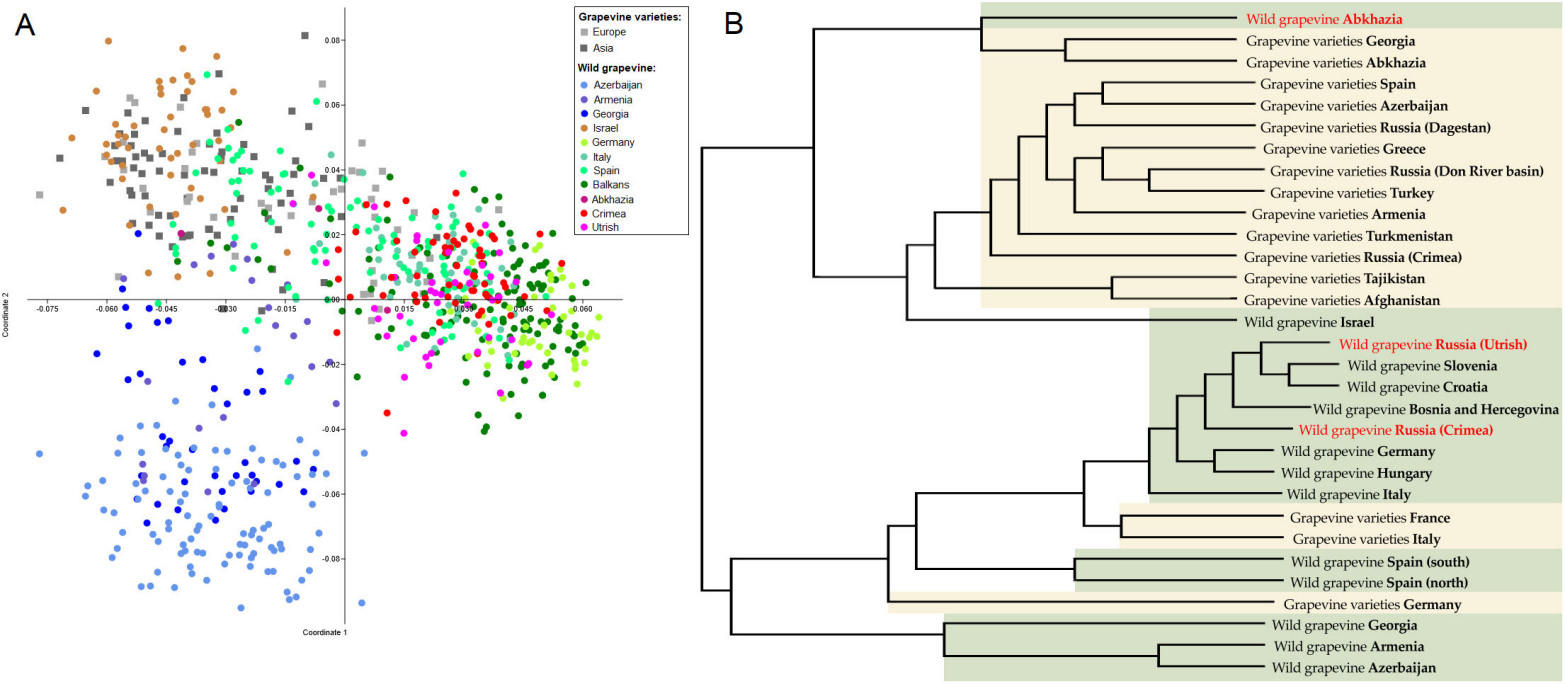
Population genetic analysis of wild grapevine from the Black Sea region in comparison with grapevine varieties and wild grapevine from different geographic regions using **(A)** the PCoA and **(B)** the UPGMA methods. On the dendrogram, wild grapevine from the Black Sea region is marked in red.

As a result of PCoA of wild and cultivated grapevines (**Supplementary Table S5**), the samples were divided into three groups (**Figure 2A**). The first group included genotypes of *V. vinifera* ssp. *sylvestris* from Transcaucasia (Azerbaijan, Armenia, Georgia). The second group comprised cultivars (of both European and Asian origin), wild grapevines from Israel, and, partly, Spain. The largest, third group included *V. vinifera* ssp. *sylvestris* from Europe, some European cultivars, and wild grapevine from the Black Sea region.

The dendrogram reflecting the hierarchical division of the sample groups in general clustered similarly to the PCoA plot (**Figure 2B**). Wild grapevine from the Crimean and Utrish nature reserves clustered together with European *V. vinifera* ssp. *sylvestris*, whereas wild grapevine from Abkhazia clustered together with native varieties from Abkhazia and Georgia.

**Figure 3:**
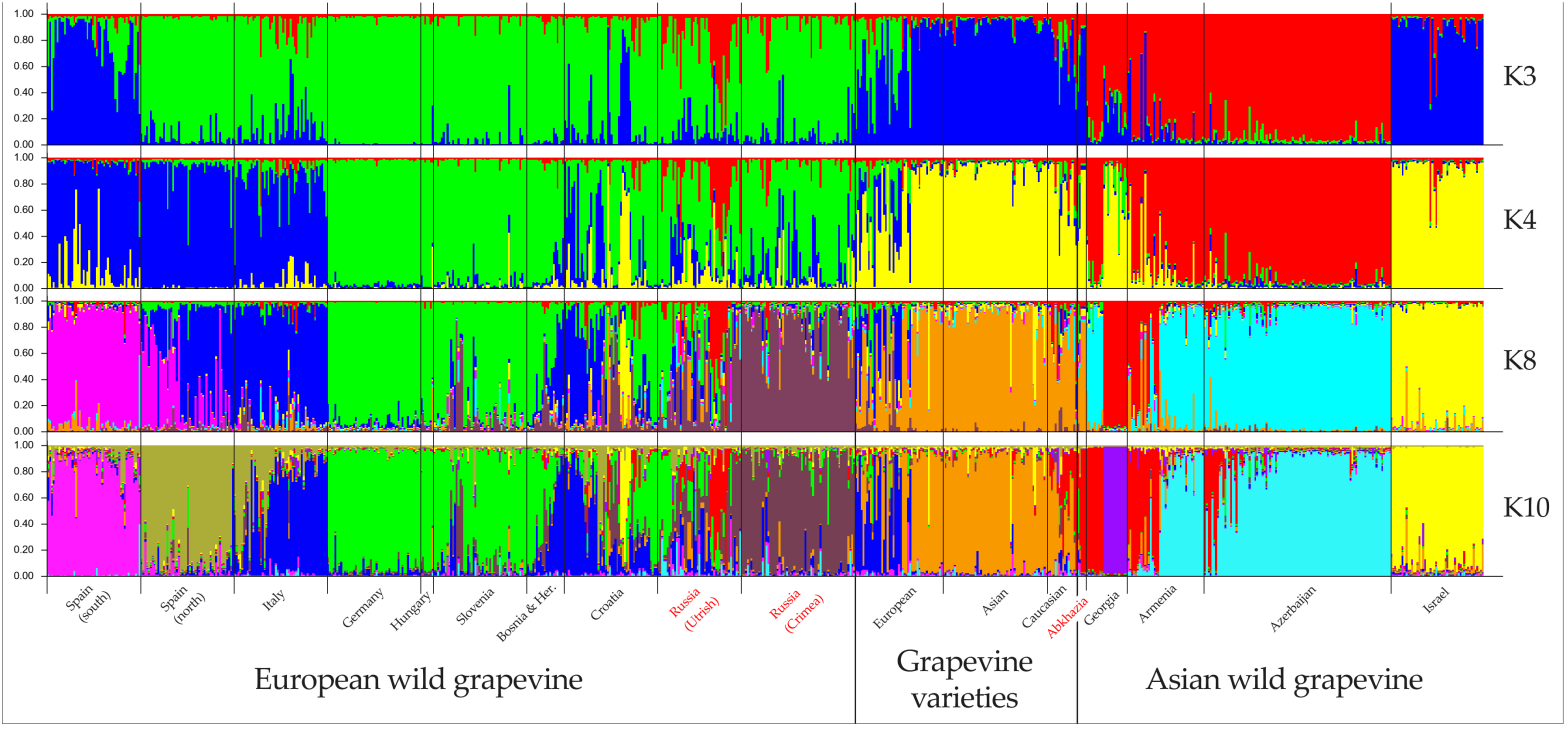
Population structure analysis of wild grapevine from different geographic regions using STRUCTURE software. Wild grapevine from the Black Sea region is marked in red.

A detailed study of the population structure of wild and cultivated grapevine was made possible by Bayesian analysis in STRUCTURE software. The optimal number of clusters for describing the population structure in the available sample selection (**Supplementary Table S5**) was three, four, eight, and ten (**Supplementary Table S10**). The analysis at K = 3 in general made it possible to divide grapevines into cultivated, wild European, and wild Asian (**Figure 3**). However, wild grapevines from Israel and southern Spain were assigned to the same cluster as cultivated grapevines. Almost every geographic population had samples distributed among different clusters. At higher K values, wild grapevines split into subgroups based on geography. The most complex structure was observed in the wild grapevine samples from Croatia and Utrish.

At K = 10, most of the wild grapevines from Crimea formed a single cluster (**Figure 3**). It also included a few samples of *V. vinifera* ssp. *sylvestris* from Slovenia, Croatia, and Utrish. The genotypes of the wild grapevine from Abkhazia belonged to a cluster that included some samples of *V. vinifera* ssp. *sylvestris* from Georgia, Armenia, Azerbaijan, and Utrish, as well as some Caucasian varieties. The samples of wild grapevine from Utrish did not form a single subgroup and belonged to clusters that included wild grapevine from both Europe and Asia.

The results of the analyses indicate that wild grapevine populations from the Crimean and Utrish nature reserves are genetically close to *V. vinifera* ssp. *sylvestris* from Europe. This observation, together with the absence of the hermaphroditism allele in most of the studied samples, allows us to attribute wild grapevine from Utrish and Crimea to *V. vinifera* ssp. *sylvestris*. The population of wild grapevine from Abkhazia was related to both the native varieties from Abkhazia and Georgia and to a number of samples of *V. vinifera* ssp. *sylvestris* from this region.

### 3.2 RNA-seq data analysis of viromes

The sequencing of 50 total RNA libraries of grapevine from the Black Sea region yielded a total of approximately 793 million reads, with an average of 16 million reads per library. Owing to preprocessing, the number of reads decreased by more than three times and, on average, comprised 5 million reads per library. The detailed sequencing and preprocessing results are provided in **Supplementary Table S11**.

A smaller number of reads were obtained from the Abkhazian samples than from the other samples; on average, there were 7 million raw reads per library and only 2.5 million reads after preprocessing. Sequencing of the Crimean samples made it possible to obtain an average of 17 million reads per library and 5.7 million after preprocessing. The data obtained from sequencing samples from the Utrish Nature Reserve were comparable to the results of sequencing the Crimean Nature Reserve samples. On average, 17 million raw reads per library were obtained; after preprocessing, their number was reduced to ~4.4 million per library. Most of the preprocessed reads in all the libraries were assigned by Kraken2 as plant reads (**Supplementary Figure S3** and **Supplementary Table S12**). Viral reads ranged from 0% to 0.14%. Some of the preprocessed reads were assigned by Kraken2 to phytopathogenic bacteria, such as *Agrobacterium tumefaciens, Allorhizobium ampelinum, Agrobacterium vitis, Pseudomonas syringae*, and several species of phytoplasmas (**Supplementary Figure S4**).

Preprocessed reads from each library were used for *de novo* assembly. Using Geneious assembler, between 20 thousand and 92 thousand contigs were assembled. Using the SPAdes assembler, from 200 to 8,000 contigs were assembled (**Supplementary Table S11**).

### 3.3 Identification of known grapevine viruses and viroid

The summarized results of read mapping, TBLASTX contig analysis, Kraken2 read classification, and PCR validation are provided in **Supplementary Table S13**. In 50 libraries, a total of ten known grapevine viruses and one viroid were identified.

Depending on the area, the composition of the identified viruses and viroids varied greatly (**Supplementary Table S14**). Only VCV was detected in all three regions, and it was the only virus found in the Abkhazian samples. Wild grapevines from all four locations of the Utrish Nature Reserve were infected with viruses, among which there were no economically significant ones: VCV, GRSPaV, GPGV, and GVT. The most diverse composition of viruses and viroids was observed in samples collected in Alushta (Crimea); along with the viruses found in Utrish, it included the economically significant GLRaV-1 and GVA, as well as grapevine-associated tymo-like virus (GaTLV), grapevine satellite virus (GV-Sat), grapevine rupestris vein feathering virus (GRVFV), grapevine red globe virus (GRGV), and grapevine yellow speckle viroid 1 (GYSVd-1; *Apscaviroid alphaflavivitis*).

**Figure 4:**
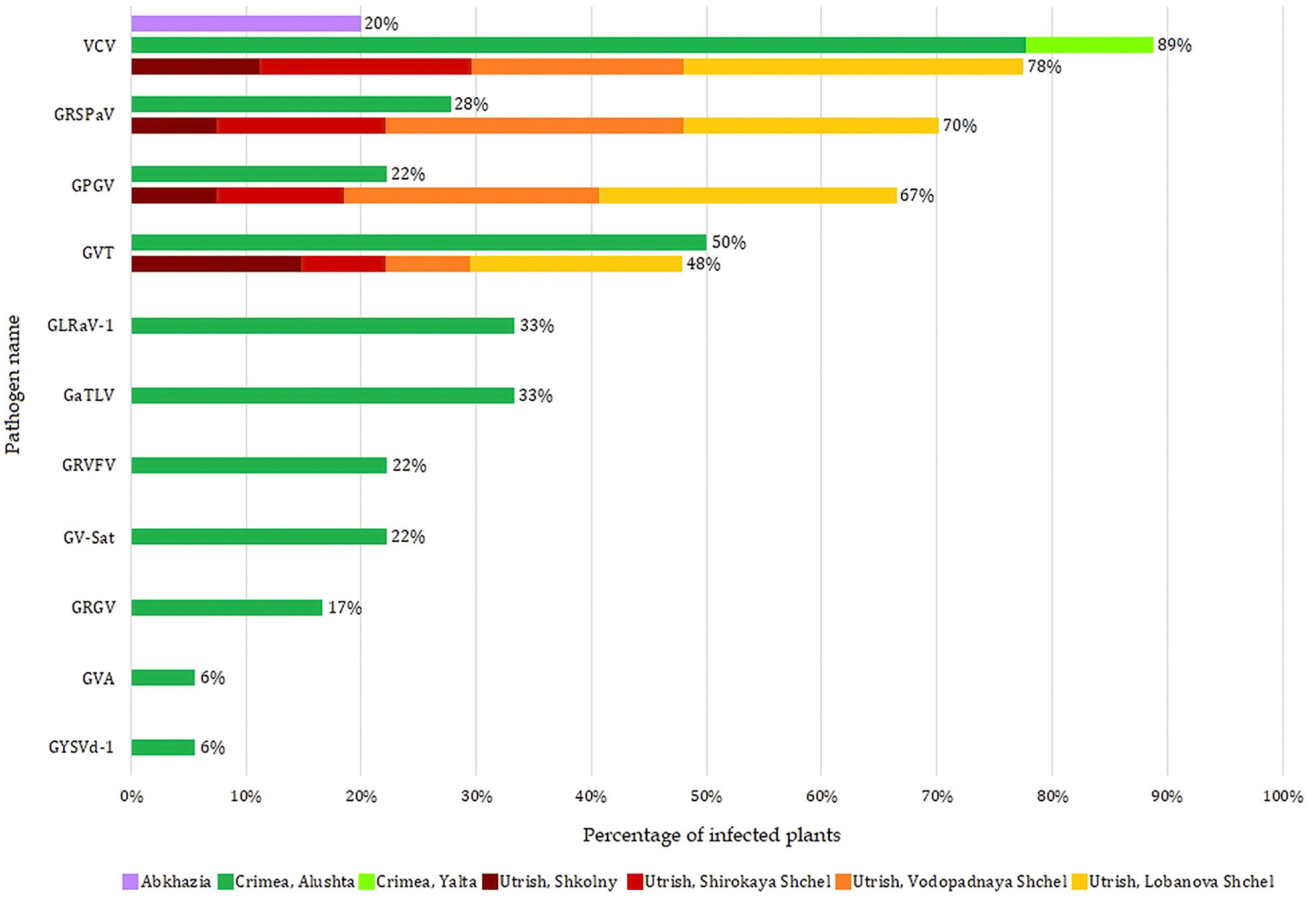
Distribution of viruses and viroid in the wild grapevines from three areas of the Black Sea region.

The most common virus in the wild grapevine samples from the Black Sea region was VCV, which infected 76% of the plants (**Figure 4**). Approximately 45% of the plants were infected with GVT, GRSPaV, and GPGV, with the latter two viruses being more common in Utrish than in Crimea.

Samples of wild grapevine were found to be infected with up to nine viruses per plant (**Supplementary Figure S5**). No viruses were found in five plants. The smallest number of preprocessed reads was found in the Abkhazian samples, and the largest was found in the Crimean samples. There was no relationship between the number of viral reads and the number of viruses detected. For example, the same number of viruses–four–was found both in a library with 2 million reads, of which fewer than 5,000 were viral, and in a library with 5.5 million reads, of which about 18 thousand were viral. Thus, the available number of reads in the libraries was sufficient for accurate virus identification.

As a result of this study, 91 complete or nearly complete virus genomes and one viroid genome were obtained, all of which were used in phylogenetic analysis (**Supplementary Table S7**). The assembly of viruses such as GRSPaV, GRVFV, GVA, GLRaV-1, and GPGV required special settings for mapping and/or selection of the optimal reference isolate (**Supplementary Table S15**). The genomes of the other viruses were assembled using read mapping to a reference genome with default settings.

Phylogenetic analysis of GLRaV-1 revealed that the isolates from libraries A14, A25, and A26 formed a phylogenetic group with two Russian isolates, whereas the isolate from library A15 clustered closer to an isolate from France (**Supplementary Figure S6**). A similar situation was observed with GV-Sat isolates: the isolates from libraries A14, A19, and A25 formed one phylogroup, whereas the isolate from library A15 was located at a distance from them (**Supplementary Figure S7**). The GYSVd-1 isolate belongs to type 3 (**Supplementary Figure S8**). The only GRVFV isolate was phylogenetically very distant from the other Russian isolates (**Supplementary Figure S9**). The GVA isolate was assigned to phylogroup I, which, to date, has not been associated with any symptoms in grapevine (**Supplementary Figure S10**) (Wu et al., 2023).

### 3.4 Discovery of novel grapevine-associated viruses

#### 3.4.1 Abrau grapevine-associated virus

Using the Geneious and SPAdes assemblers, a 7,098 bp contig was assembled in library A51 (sample B1). A TBLASTX analysis against the NCBI reference viral genome database revealed the similarity of this contig with rice stripe necrosis virus (*Benyvirus oryzae*) RNA1 NC_038775 (*E*-value = 8.99 × 10^−43^, 30.8% identical sites). A BLASTN analysis of the contig against the GenBank nucleotide database revealed no matches with available sequences, but a BLASTX analysis against the same database revealed similarities of the contig with RNA1 sequences of viruses from the family *Benyviridae* (26.3%–35.9% identical sites). This suggested that the contig belongs to the genome of a novel virus, tentatively named Abrau grapevine-associated virus (AGaV).

A total of 411 reads from library A51 were mapped to this contig, with an average coverage of 7.8. Reads from other libraries were not mapped to this contig. The 5’- and 3’-UTRs of the novel virus were sequenced using the Step-Out RACE. The complete nucleotide sequence of AGaV is 7,150 nt in length. PCR with the contig-specific primers beny_920F/beny_1329R (**Supplementary Table S2**) revealed that AGaV was detected only in sample B1.

Because benyviruses have a multipartite genome (Gilmer and Ratti, 2017), we expected to find contigs related to other possible AGaV RNAs. A local database containing 2,034 nucleotide sequences of viruses from the family *Benyviridae* was constructed based on sequences from GenBank. As a result of TBLASTX analysis of contigs against this database, no new AGaV contigs were found. To detect other AGaV RNAs, we prepared and sequenced two new libraries from the sample B1. One library was constructed from the original total RNA, and the other was constructed from re-extracted total RNA. The two new libraries were analyzed using the same procedure used for A51, but no new AGaV contigs were detected. These findings suggest that the AGaV genome is represented by a single RNA.

**Figure 5:**
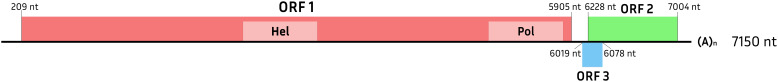
Putative genome organization of Abrau grapevine-associated virus. Open reading frames are shown by colored rectangles. Light colors indicate conserved domains predicted by InterPro.

The AGaV RNA was predicted to have three open reading frames (**Figure 5**). ORF1 encodes a 1,898 aa polyprotein containing the (+) RNA virus helicase core domain and the catalytic core domain of RdRp in the family *Benyviridae* (according to InterPro). ORF2 encodes a 308 aa protein of unknown function. ORF3 partially overlaps with ORF2 and presumably encodes a 69 aa protein.

**Figure 6:**
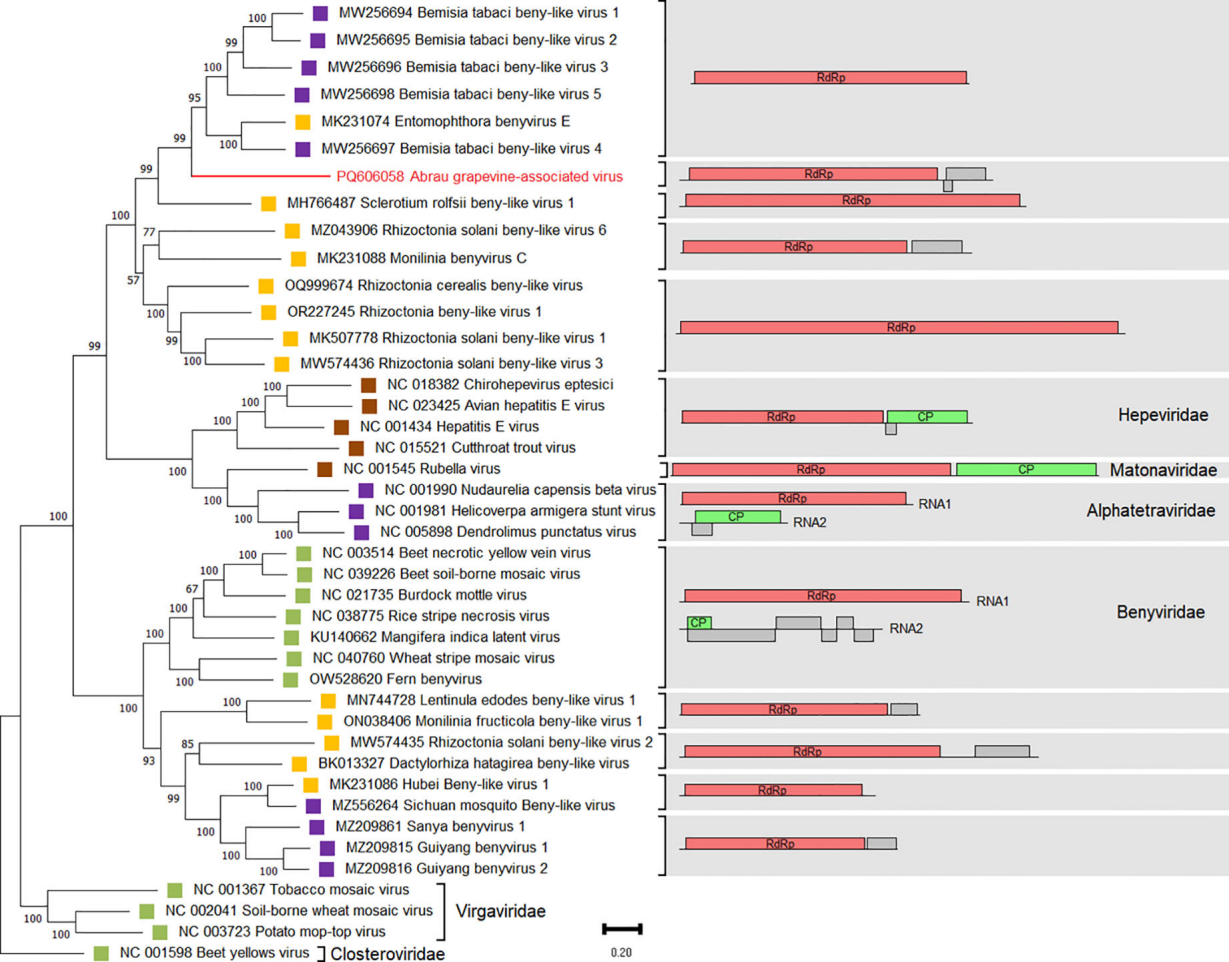
Phylogenetic analysis based on nucleotide sequences of ORF1 encoding the RdRp of Abrau grapevine-associated virus (AGaV) and members of the order *Hepelivirales*. The right panel shows the genome organizations of these viruses. The name of AGaV is highlighted in red. Host specificity is indicated by colored squares: violet – arthropods; yellow – fungi; brown – vertebrates; green – plants. The tree was constructed in MEGA11 using the maximum likelihood method and GTR model with 1,000 bootstrap replicates. Members of the families *Virgaviridae* and *Closteroviridae* were used as an outgroup.

Nucleotide sequences of ORF1 encoding a replication-associated polyprotein of viruses from the order *Hepelivirales* were used for phylogenetic analysis of AGaV. Viruses of the order *Martellivirales* (*Virgaviridae* and *Closteroviridae*) were used as an outgroup. As a result, AGaV clustered with beny-like viruses associated with fungi or insects (**Figure 6**). Similar to AGaV, they were found to comprise only one RNA of 6,000–12,000 nt in length, containing one or two ORFs.

An SDT analysis revealed that the amino acid sequences of the replication-associated proteins of AGaV and the viruses closest in phylogeny had a similarity of 27.3%–34.7% (**Supplementary Table S16**). Moreover, the similarity between the replication-associated proteins of AGaV and viruses from the genera *Benyvirus* and *Hepevirus* was 23.8%–24.9% and 18.7%–21.2%, respectively.

#### 3.4.2 Taurida grapevine-associated virus

Using the Geneious assembler, two contigs, 965 bp and 1,504 bp in length, were assembled from library A10. A TBLASTX analysis showed their similarity to cherry virus Trakiya (CVT) NC_040561 of the order *Picornavirales* (E-value = 7.97 × 10^−59^, 44.2% identical sites, and 1.52 × 10^−110^, 47.3%, respectively). A BLASTN search against the GenBank nucleotide collection did not reveal any matches with the sequences available in the database. This suggested that the discovered contigs belonged to a novel virus, tentatively named Taurida grapevine-associated virus (TGaV).

Reads from other libraries were mapped to both contigs. In seven of them, the mapping was successful, which allowed us to extend the contigs to 1,800 bp and 2,457 bp. All preprocessed reads from these seven libraries were combined into one pool, and reads that mapped to the *V. vinifera* genome were removed. The remaining reads were used for *de novo* assembly. The assembled contigs were analyzed using TBLASTX against a local database that included 5,570 nucleotide sequences of the order *Picornavirales*. As a result, we discovered one more contig homologous to CVT, which was 766 bp long.

The three discovered contigs were aligned to the 5’ terminal, central, and 3’ terminal regions of the CVT genome. To assemble the complete TGaV genome, we selected specific primers for the contigs and performed Sanger sequencing (**Supplementary Table S2**). The terminal regions of the genome were determined using the Step-Out RACE. As a result, the whole-genome sequence of TGaV with a length of 8,547 nt was obtained. PCR validation confirmed the presence of TGaV in eight samples of wild grapevine from Alushta (Crimea) (**Supplementary Table S13**).

**Figure 7:**
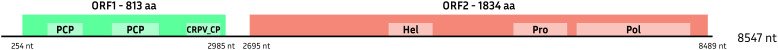
Putative genome organization of Taurida grapevine-associated virus. Open reading frames are shown by colored rectangles. Light colors indicate conserved domains predicted by InterPro.

The TGaV genomic RNA was predicted to have two open reading frames (**Figure 7**). ORF1 encodes a structural polyprotein 813 aa long with three conserved domains: two corresponding to the picornavirus capsid protein and one to the CRPV capsid protein (according to InterPro). ORF2 encodes a nonstructural polyprotein 1,834 aa long with conserved domains typical of *Picornavirales*: an RNA helicase, a 3C/3C-like protease, and an RNA-dependent RNA polymerase (Hel, Pro, and Pol, respectively).

**Figure 8:**
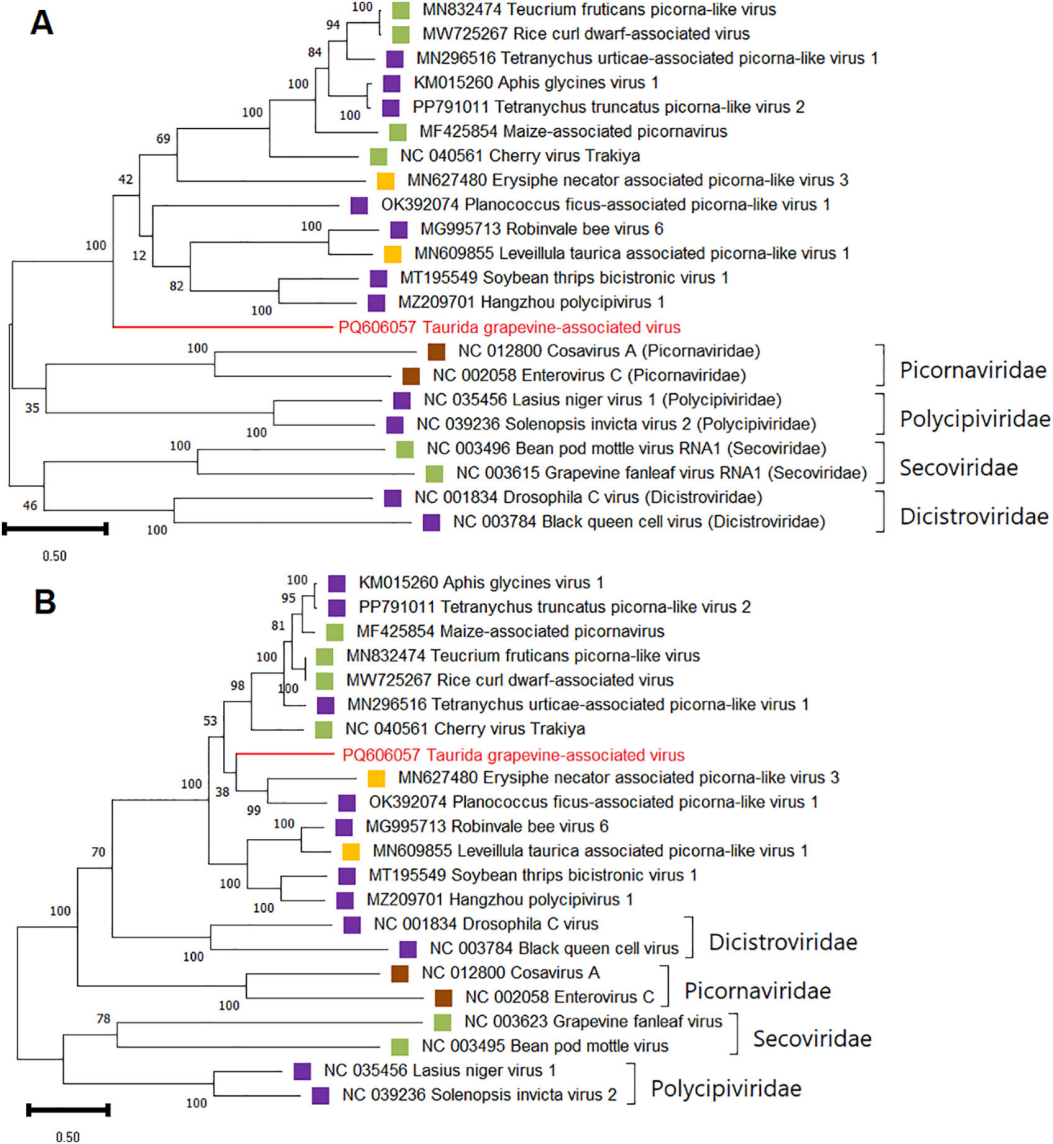
Phylogenetic analysis based on amino acid sequences of **(A)** the RdRp and **(B)** the CP of Taurida grapevine-associated virus (TGaV) and members of the order *Picornavirales*. The name of TGaV is highlighted in red. Host specificity is indicated by colored squares: violet – arthropods; yellow – fungi; brown – vertebrates; green – plants. The tree was constructed in MEGA11 using the maximum likelihood method and LG model with 1000 bootstrap replicates.

For phylogenetic and SDT analyzes of TGaV, amino acid sequences of replication-associated and coat proteins of viruses of the order *Picornavirales* (families *Picornaviridae, Polycipiviridae, Dicistroviridae, Secoviridae*, and unclassified *Picornavirales*) were used. In both dendrograms, TGaV most closely clustered with a group of monopartite bicistronic unclassified picorna-like viruses (**Figure 8**). This suggests that TGaV also has a monopartite genome.

An SDT analysis revealed that the replication-associated polyprotein of TGaV is 22%–26% identical to the corresponding polyproteins of viruses from the families *Picornaviridae*, *Polycipiviridae*, *Dicistroviridae*, and *Secoviridae* and 28.8%–33% identical to the polyproteins of phylogenetically similar unclassified picorna-like viruses (**Supplementary Table S17**). The identity of the coat proteins of TGaV and viruses from the above families was 19.4%–25.6%, whereas the identity of TGaV and phylogenetically related viruses was 43.1%–48.3% (**Supplementary Table S18**).

### 3.5 Population genetic analysis of the most prevalent viruses

#### 3.5.1 Vitis cryptic virus

**Figure 9:**
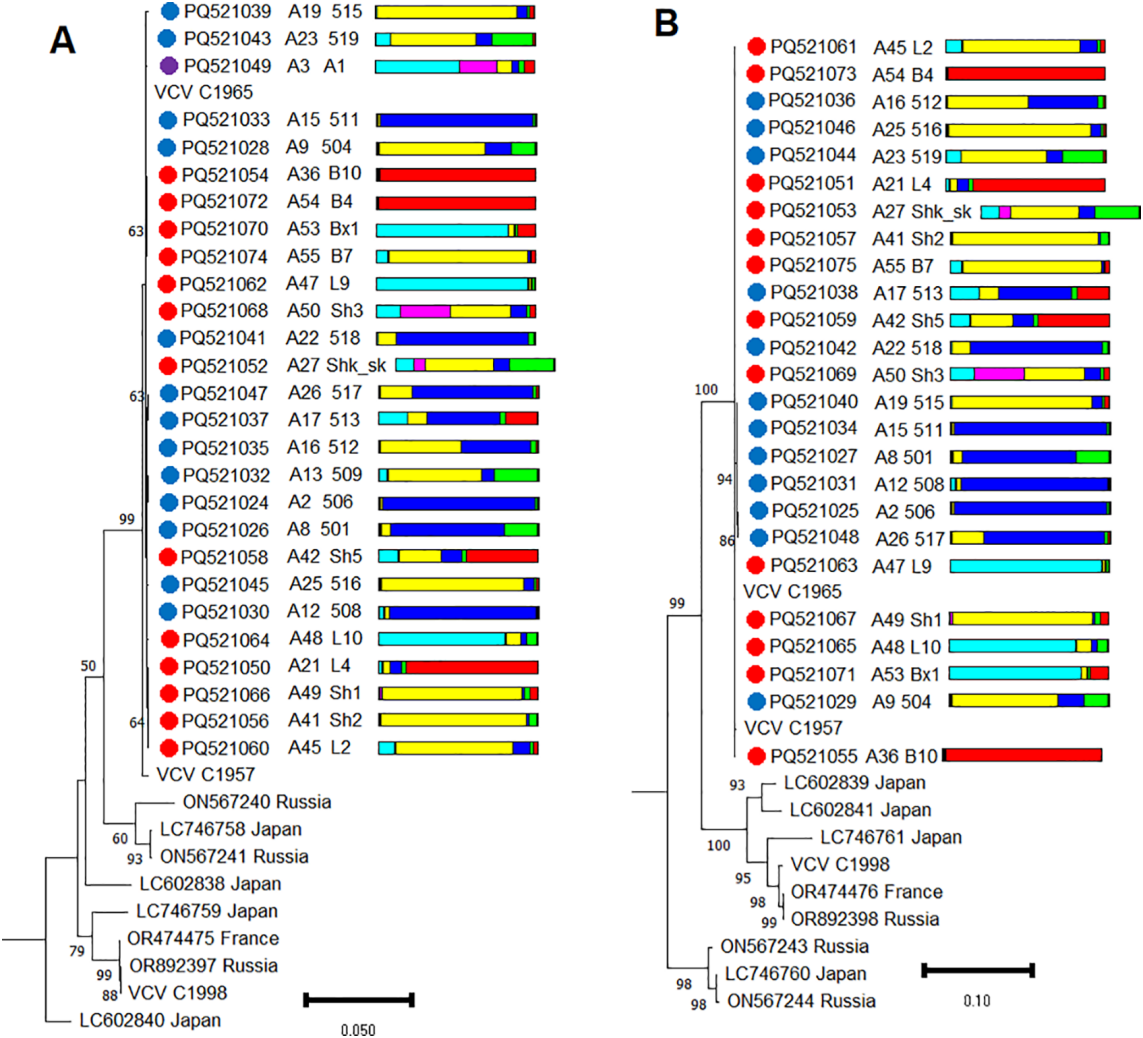
Phylogenetic analysis based on complete genome sequences of **(A)** RNA1 and **(B)** RNA2 of Vitis cryptic virus (VCV) isolates obtained from Abkhazia (violet dot), Crimea (blue dots), Utrish (red dots) and world isolates. The colored bars indicate the belonging of the host plant to the genetic cluster based on the results of population structure analysis (**Figure 1B**). The tree was constructed in MEGA11 using the maximum likelihood method, T92 (for RNA1) and TN93 (for RNA2) models with 1000 bootstrap replicates.

A recombinant analysis of VCV isolates from the wild grapevine from the Black Sea region did not detect any recombination events. For population genetic analysis of VCV, we used the complete nucleotide sequences of RNA1 and RNA2 obtained in this study and downloaded from GenBank. All VCV isolates from the Black Sea region with high bootstrap support formed a separate clade (**Figure 9**), which also included two isolates from the Magarach ampelographic collection (Crimea).

Multiple alignments of VCV isolates revealed that all currently available genomes are characterized by high sequence identity (94.3%–100% identical sites for RNA1 and 85.9%–100% for RNA2). Moreover, within the population from the Black Sea region, the identity was as high as 99.5%–100%.

The nucleotide diversity index π for all available VCV genomes was 0.0176 (RNA1) and 0.0359 (RNA2) (**Supplementary Table S19**), whereas for the population from the Black Sea region it was 0.0013 and 0.0011, respectively. Tajima’s neutrality test was statistically significant only for the VCV population from the wild grapevine from the Black Sea region (RNA1) and had a value of -1.520, which may indicate purifying selection (**Supplementary Table S19**).

The fixation index Φst for the VCV population from the wild grapevine from the Black Sea region versus all available in the GenBank isolates was 0.678 for RNA1 and 0.734 for RNA2, which indicates a high level of genetic differentiation (**Supplementary Table S20**).

#### 3.5.2 Grapevine virus T

**Figure 10:**
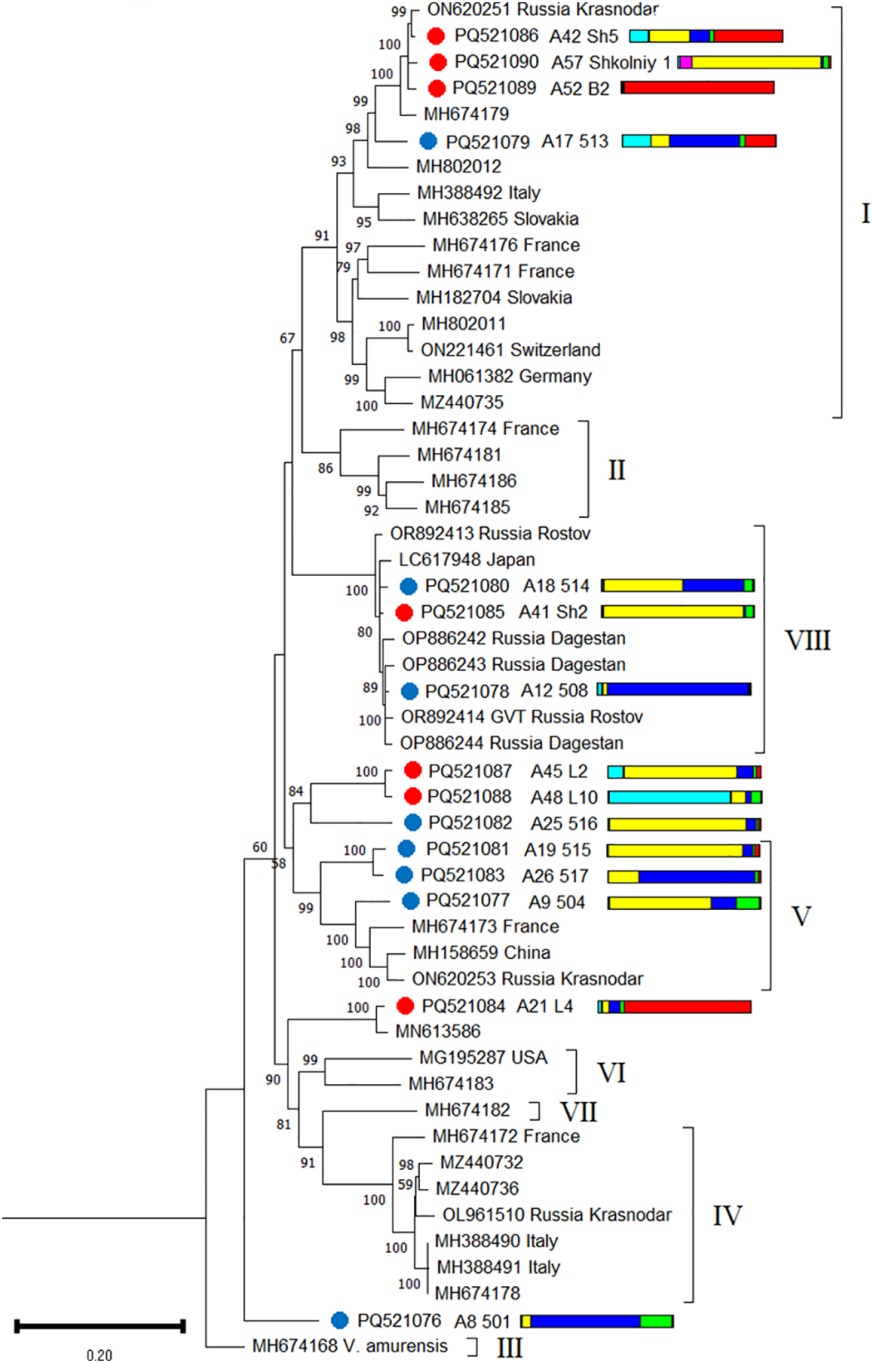
Phylogenetic analysis based on complete genome sequences of grapevine virus T (GVT) isolates obtained from Crimea (blue dots), Utrish (red dots) and world isolates. The colored bars indicate the belonging of the host plant to the genetic cluster based on the results of population structure analysis (**Figure 1B**). The tree was constructed in MEGA11 using the maximum likelihood method and GTR model with 1000 bootstrap replicates.

Among the GVT isolates discovered in this study, two were recombinant: 501 and 517 (**Supplementary Table S21**). A phylogenetic analysis revealed that the GVT isolates from the Black Sea region belonged to phylogroups I, V, and VIII. Another five isolates clustered outside existing phylogroups (**Figure 10**). Phylogenetically close isolates were detected in grapevines belonging to different genetic clusters.

For population genetic analysis, we selected only complete genomes of GVT from Europe and Russia as the two most represented populations in GenBank. The nucleotide diversity index of GVT varied from 0.118 (Russian population) to 0.157 (European population), and for isolates from the grapevines in the Black Sea region, it was 0.153 (**Supplementary Table S19**). Tajima’s neutrality test for the three populations was statistically insignificant. Statistically significant values of the Φst index were obtained for populations from Europe and the Black Sea region (Φst = 0.055), as well as for populations from Europe and Russia (Φst = 0.155) (**Supplementary Table S20**).

#### 3.5.3 Grapevine rupestris stem pitting–associated virus

**Figure 11:**
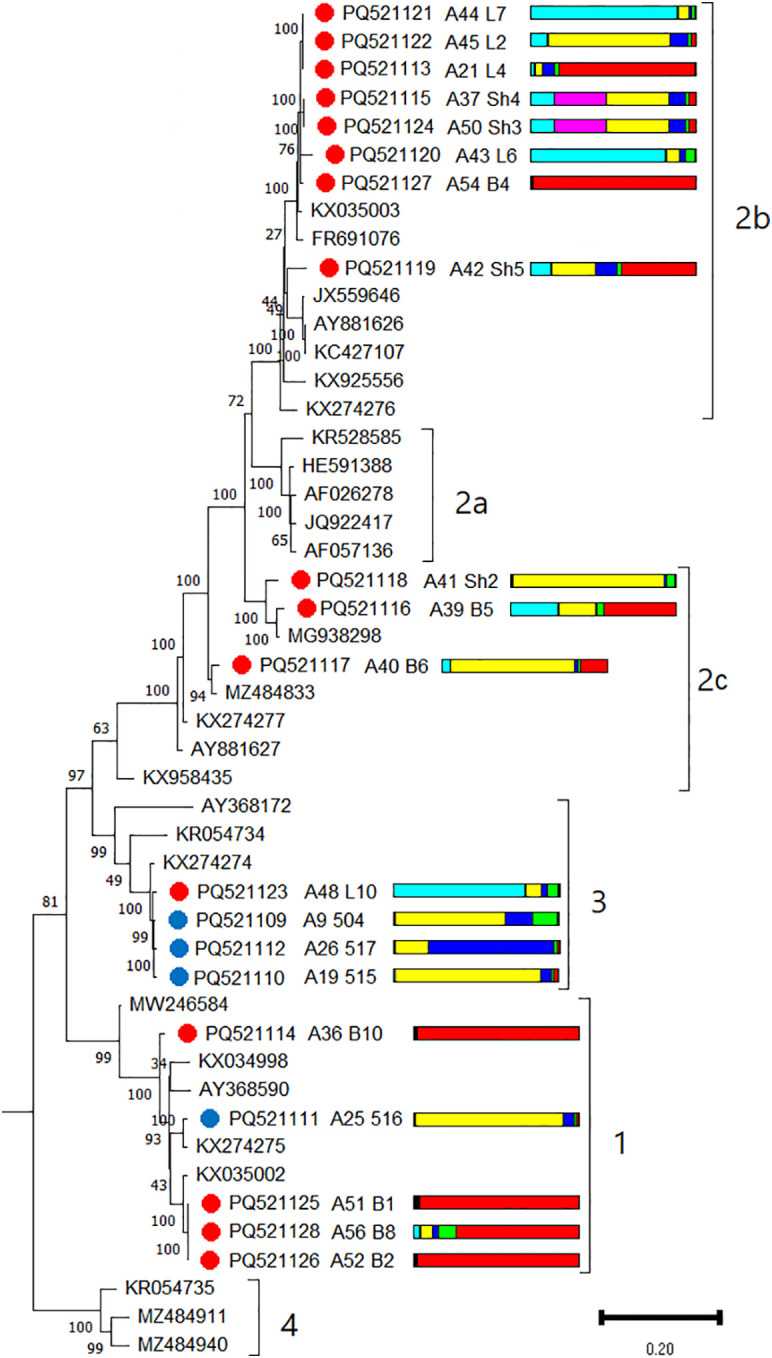
Phylogenetic analysis based on complete genome sequences of grapevine rupestris stem pitting–associated virus (GRSPaV) isolates obtained from Crimea (blue dots), Utrish (red dots) and representative phylogroup members. The colored bars indicate the belonging of the host plant to the genetic cluster based on the results of population structure analysis (**Figure 1B**). The tree was constructed in MEGA11 using the maximum likelihood method and GTR model with 1000 bootstrap replicates.

A recombinant analysis revealed the presence of one recombinant (L6) among the 20 GRSPaV isolates identified in this study (**Supplementary Table S21**). For phylogenetic analysis, isolates from the wild grapevine from the Black Sea region were compared with representative members of phylogroups 1, 2a, 2b, 2c, 3, and 4 (Hily et al., 2018). Isolates from Crimea belonged to phylogroups 1 and 3, whereas isolates from Utrish belonged to 1, 2b, 2c, and 3 (**Figure 11**). Notably, phylogroup 2c was divided into four separate branches, which probably indicates the need to distinguish new groups while accounting for the identified novel GRSPaV genomes. All GRSPaV isolates from phylogroup 2b identified in this study were detected in the wild grapevine of the Utrish State Nature Reserve; however, the host plants belonged to different genetic clusters. Even within the same location, phylogenetically close isolates (L2, L4, L6, and L7) were found in grapevines assigned to different genetic clusters. The same observation was made for other phylogroups of GRSPaV isolates from the wild grapevine from the Black Sea region.

Isolates from North America, Europe, Asia, and Russia were used for population genetic analysis. The nucleotide diversity index π for the GRSPaV population from the Black Sea region was 0.163 (**Supplementary Table S19**), which is comparable to the overall value for all GRSPaV isolates used in the analysis (π = 0.17). The most genetically diverse populations were from Asia (π = 0.193) and North America (π = 0.184). The π index for the European population, the largest in this study, was 0.17. Among the populations considered, the lowest genetic diversity index was observed in the GRSPaV population from Russia (0.153).

Tajima’s neutrality test of these samples revealed low statistical significance. The Φst value for all GRSPaV populations under consideration did not exceed 0.076, which indicates a high level of gene flow between them (**Supplementary Table S20**). No statistically significant Φst values were obtained for the North American population. Thus, the GRSPaV population from the wild grapevine from the Black Sea region has close genetic connections with GRSPaV isolates around the world.

#### 3.5.4 Grapevine pinot gris virus

**Figure 12:**
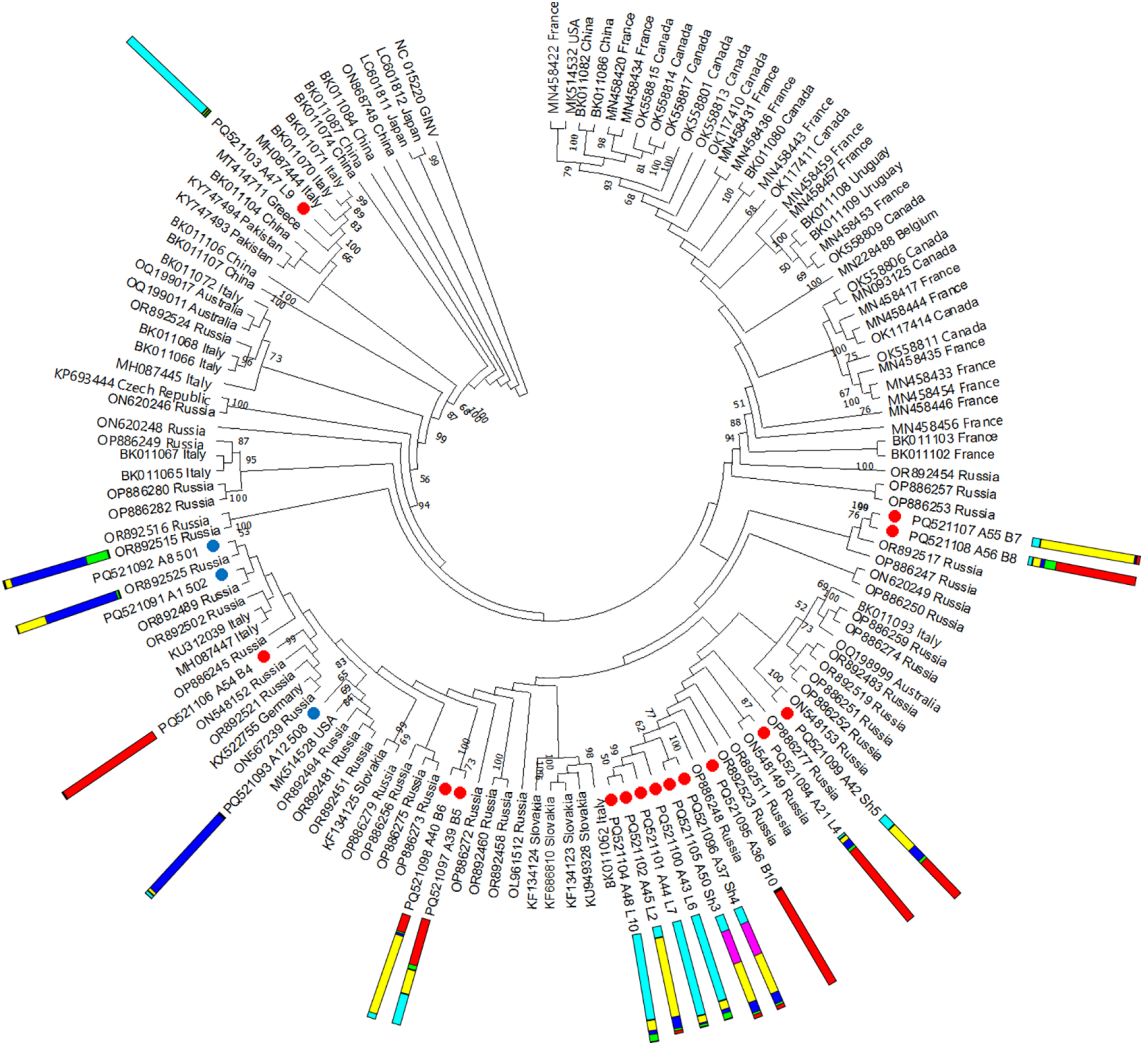
Phylogenetic analysis based on complete genome sequences of grapevine pinot gris virus (GPGV) isolates obtained from Crimea (blue dots), Utrish (red dots) and world isolates. The colored bars indicate the belonging of the host plant to the genetic cluster based on the results of population structure analysis (**Figure 1B**). The tree was constructed in MEGA11 using the maximum likelihood method and GTR model with 1000 bootstrap replicates.

No recombinants were detected among the 18 GPGV isolates identified in this study. Phylogenetic analysis revealed that most isolates from the wild grapevine from the Black Sea region are grouped together and are close to isolates from Russia, Italy, Germany, and Slovakia (**Figure 12**). Only one isolate from this study was phylogenetically distant from the others and clustered with isolates from Greece and Italy. In most cases, isolates found in grapevines from the same location clustered close to each other, regardless of which genetic cluster the host plant belonged to.

The nucleotide diversity index for the entire set of GPGV genomes was 0.028 (**Supplementary Table S19**). The greatest diversity was noted for the population from Asia (π = 0.066); for the remaining studied populations, the index varied from 0.015 to 0.026. The D value of Tajima’s statistic for all populations was negative, from −1.75 for the North American population to −2.32 for the Russian population, which may indicate purifying selection (**Supplementary Table S19**).

The pairwise Φst values revealed that the GPGV population from the wild grapevine of the Black Sea region is genetically close to the population from cultivated grapevine in Russia (Φst = 0.036) and, to a lesser extent, to the population from Europe (Φst = 0.085) (**Supplementary Table S20**). GPGV populations from Asia, Australia, and North America exchange genes less frequently with populations from the Black Sea region, with Φst values of 0.199, 0.169, and 0.223, respectively.

It is known that at the end of the reading frame encoding the MP, some GPGV strains have a polymorphism leading to the formation of an early stop codon (Saldarelli et al., 2015). This polymorphism is associated with the manifestation of grapevine leaf mottling and deformation (Bertazzon et al., 2017). The GPGV isolates obtained in this study did not contain an early stop codon at this position (**Supplementary Figure S11**).

## 4 Discussion

### 4.1 Place of wild grapevine from the Black Sea region in the grapevine gene pool

The results of the population genetic analysis of grapevines from different geographical regions obtained in this study indicate a high level of genetic differentiation between Asian and European populations of *V. vinifera* ssp. *sylvestris*. Similar results were reported in a recent study of wild grapevine in Slovenia (Perko et al., 2024). Many European, Abkhazian, and Georgian varieties were genetically close to the wild grapevine of their regions. This confirms the assumption of the influence of wild forest grapevine on the formation of the gene pool of cultivated grapevine in Europe (Magris et al., 2021) and is consistent with the presence of a center of grapevine domestication in the Caucasus along with the West Asian center (Dong et al., 2023).

Populations of wild grapevine from the Crimean and Utrish nature reserves are genetically isolated from most *V. vinifera* ssp. *vinifera* cultivars and are genetically related to *V. vinifera* ssp. *sylvestris* from southeastern Europe. The samples from the Utrish Nature Reserve were distinguished by a complex population structure and were grouped together with wild grapevine from both Crimea and Abkhazia. This is probably because the Utrish Nature Reserve is located on the Black Sea coast between Crimea and Abkhazia. Moreover, wild grapevines from Crimea are not genetically related to the native Crimean varieties, which means that they represent geneplasm introduced from outside. This finding is consistent with previously obtained data on the origin of native Crimean varieties through introduction from other regions (Sekridova et al., 2022).

A different situation was observed in the analysis of wild grapevine samples from Abkhazia. A high level of gene flow between them and local native varieties, as well as genetic isolation from other groups of cultivated and wild grapevine, indicates that this population is represented by forms related to local varieties and a few samples of Caucasian *V. vinifera* ssp. *sylvestris*. An earlier study of grapevines in this geographical region also revealed a genetic relationship between wild forms and native varieties (Imazio et al., 2013; Ekhvaia et al., 2014; Riaz et al., 2018).

Thus, the population genetic analysis of wild grapevine from different areas of the Black Sea region that we performed made it possible for the first time to determine its place in the world gene pool in comparison with cultivated and wild grapevine from different geographical regions.

### 4.2 Viral populations of wild grapevine from the Black Sea region

Recently, Haegeman et al. demonstrated that the Kraken2 metagenomic classifier can be applied to RNA-seq data for rapid analysis of preprocessed reads and detection of cellular organisms. We applied this method to our data and tested Kraken2 for virus detection (Haegeman et al., 2023). For some viruses (GPGV, GaTLV, and GV-Sat), the number of reads classified by Kraken2 was comparable to the number of reads mapped to the reference genome in Geneious Prime software (**Supplementary Table S13**). Approximately two times fewer GRGV, GRVFV, and GLRaV-1 reads were classified by Kraken2 than were mapped to the reference genomes. For the detection of GRSPaV and GVA, the number of reads mapped to RefSeq was several orders of magnitude greater than the number of reads classified by Kraken2. Nonetheless, when the genome of the detected virus was present in the database used by Kraken2, its reads were identified in all virus-positive samples.

Compared with commercial and collection vineyards in southern Russia (Navrotskaya et al., 2021; Shvets et al., 2022a, 2022b; Belkina et al., 2023), wild grapevines from the Black Sea region were generally infected with a small number of viruses. The number of viruses per plant averaged three (**Supplementary Figure S5**), whereas in cultivated grapevine from southern Russia, an average of 5–10 viruses per plant were detected. Moreover, in this study, no virus was detected in five plants, four of which grew in Abkhazia.

In the wild grapevine from Abkhazia, which is genetically distinct from the grapevine from the Crimean and Utrish nature reserves, only one virus (VCV) was detected, which limits our ability to conduct population genetic analysis of grapevine viruses from all three study areas. The almost complete absence of viral infections in the wild grapevine from Abkhazia is interesting and unusual; further studies will help to shed light on the virome of *V. vinifera* ssp. *sylvestris* in this area.

**Figure 13:**
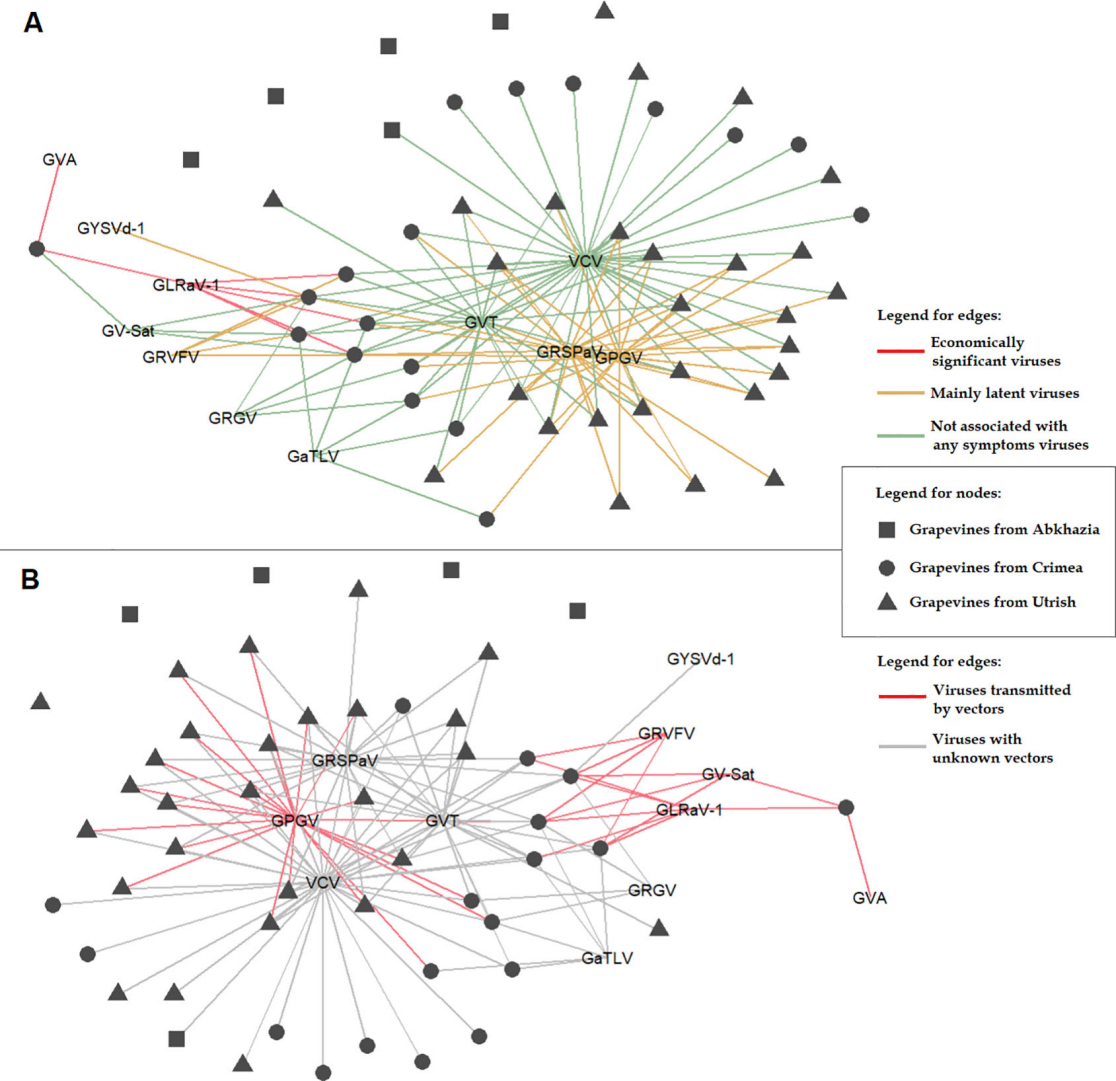
Associations between wild grapevines from the Black Sea region and the detected viruses, showing **(A)** the economic significance of the viruses and **(B)** information on their biological vectors.

Grapevines from the other two areas were predominantly infected with VCV, GVT, GRSPaV, and GPGV (**Supplementary Table S14**). The distribution and population genetics of these viruses are discussed in detail later in this article. In the wild grapevine of the Crimean Nature Reserve, we detected, along with the four viruses mentioned above, GRVFV (22%), GRGV (17%), GV-Sat (22%), GaTLV (33%), GYSVd-1 (6%), and two economically significant viruses: GLRaV-1 (33%) and GVA (6%) (**Figure 13A**). These viruses were previously identified on *V. vinifera* ssp. *sylvestris* in Tuscany (Italy): GLRaV-1 infected 11% of the analyzed grapevines, and GVA infected 7% (Sabella et al., 2018). Phylogenetically, GLRaV-1 isolates from the Crimean Nature Reserve grapevines clustered with Russian isolates from the Anapa ampelographic collection and, with a high bootstrap, formed a separate clade (**Supplementary Figure S6**). GLRaV-1 and GVA are known to be transmitted by mealybugs (**Figure 13B**), and most other viruses found in Crimean grapevine also have known vectors (Fuchs, 2020). Notably, the viticulture industry is well developed on the Crimean Peninsula, and the planting material is usually imported from European countries. Therefore, it is likely that the listed viruses spread to the Crimean Nature Reserve grapevine from nearby commercial vineyards. Phylogenetic analysis of the isolates of these viruses found in this study demonstrated the absence of unique phylogroups specific to wild grapevine from the Black Sea region.

Only some of the viruses found in wild grapevine from the Black Sea region are associated with grapevine diseases (**Figure 13A**). The most dangerous pathogens are GLRaV-1, a causative agent of grapevine leafroll disease, and GVA, a causative agent of rugose wood disease and possibly Shiraz disease. The manifestation of these and other viral diseases occurs only when a number of factors are combined, such as a virulent genetic variant of the virus, a susceptible grapevine variety, and specific environmental conditions (Meng et al., 2017; Fuchs, 2020). Viruses such as GRSPaV, GPGV, and GRVFV are associated mainly with latent infections, but under certain conditions, they can cause rupestris stem pitting (RSP), grapevine leaf mottling and deformation (GLMD), and vein feathering disease, respectively. The other viruses detected in this study (VCV, GVT, GaTLV, GV-Sat, and GRGV) are currently not associated with the manifestation of any symptoms in grapevine. This may be due to insufficient studies and difficulty in identifying symptoms in mixed infections. However, the possibility that these viruses do not have any negative effects on grapevines cannot be excluded.

The geographic region of this study is part of the natural range of distribution of wild grapevine (Grassi and De Lorenzis, 2021). Moreover, most of the samples were collected in specially protected natural areas; therefore, these ecosystems can be expected to be minimally affected by human activity. Viruses that are widespread in wild grapevine from the Black Sea region undoubtedly play a role in the ecosystem. Apparently, they do not interfere with the growth of the studied grapevines, which have been growing in this area for decades. It is possible that certain viruses are widespread in wild grapevines precisely because they provide the host plant with a competitive advantage for survival. GRSPaV-infected grapevines under greenhouse conditions were previously shown to resist drought more effectively than uninfected plants did (Pantaleo et al., 2016). In addition, viruses from the family *Partitivitidae*, which includes VCV, are known as harmless persistent plant viruses, and some of them are capable of repelling herbivorous insects (Safari et al., 2019).

Thus, in some cases, viral infections can increase the ability of the host plant to withstand abiotic and biotic stresses. This advantage can be very beneficial in intraspecies competition and can lead to the survival of grapevines that are infected with mutualist viruses. This may be the reason why some viruses have been evolutionarily preserved in the Black Sea grapevine population. However, this issue requires in-depth study, since different viruses can adhere to different strategies of interaction with the host plant (Lefeuvre et al., 2019; Rodríguez-Nevado et al., 2020), and their widespread distribution in the wild grapevine population does not in itself imply a positive effect.

### 4.3 Population genetic analysis of the most widespread viruses in the wild grapevine from the Black Sea region

**Figure 14:**
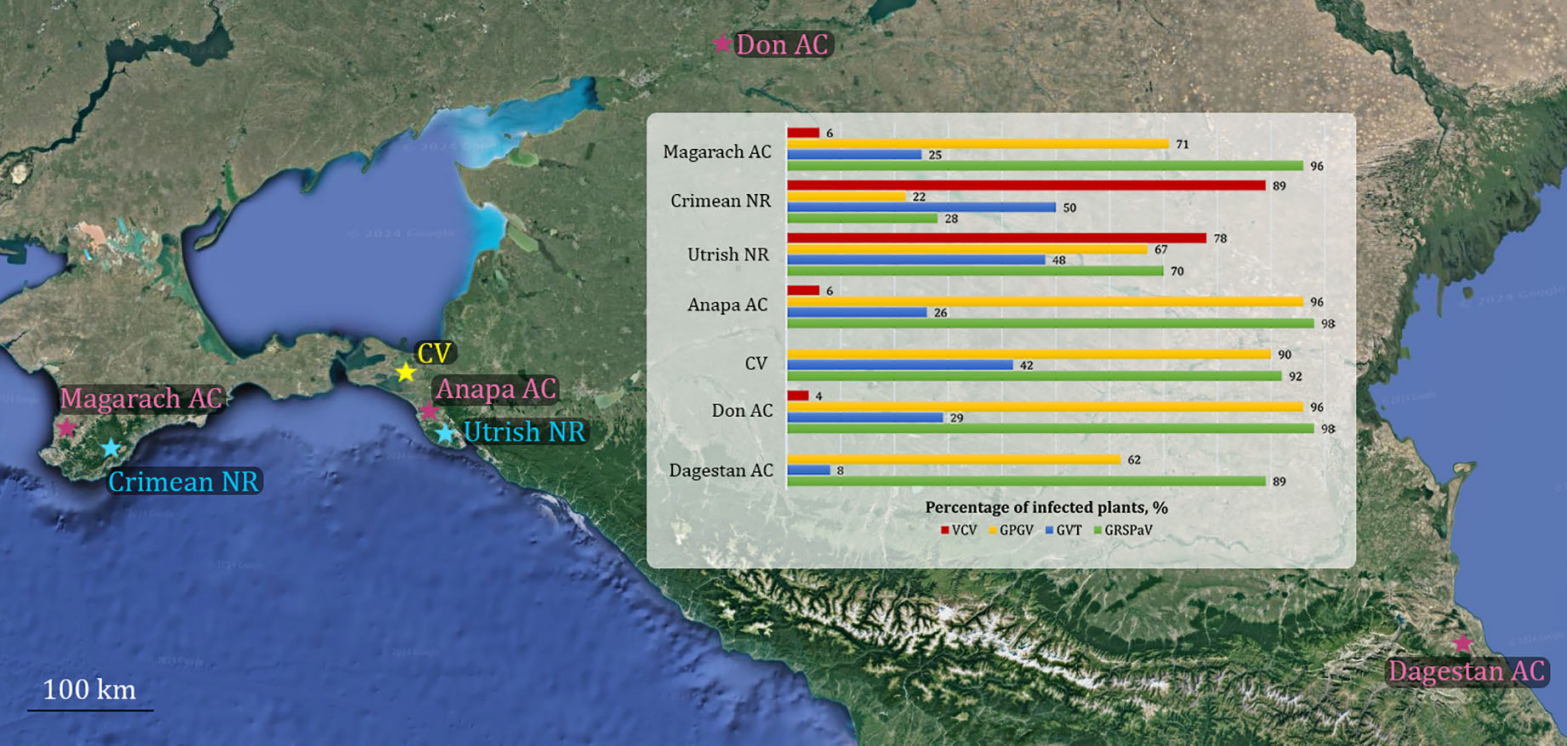
Distribution of Vitis cryptic virus (VCV), grapevine pinot gris virus (GPGV), grapevine virus T (GVT), and grapevine rupestris stem pitting–associated virus (GRSPaV) in wild grapevine from the Black Sea region and cultivated grapevine from southern Russia. Ampelographic Collections (AC) are indicated in pink, Nature Reserves (NR) in blue, and commercial vineyards (CV) in yellow.

This study revealed that VCV, GPGV, GVT, and GRSPaV are very common in the wild grapevine from the Black Sea region. To determine how typical this situation is for this region and to trace the routes of virus migration between cultivated and wild grapevine, we used the results of metavirome studies of commercial vineyards (Navrotskaya et al., 2021) and ampelographic collections (Shvets et al., 2022a, 2022b; Belkina et al., 2023) from southern regions of Russia (**Figure 14**). Notably, the Anapa ampelographic collection is located at a distance of approximately 25 km from the Utrish State Nature Reserve, where we collected some of the samples for this study, whereas the Magarach ampelographic collection is located on the Crimean Peninsula at a distance of approximately 50 km from the Crimean Nature Reserve.

#### 4.3.1 Vitis cryptic virus

One of the specific features of wild grapevine from the Black Sea region was the widespread distribution of VCV. This virus infected grapevines in all seven locations (**Figure 4**).

The population genetics of VCV have not been previously studied; however, there are data on populations of related viruses of the family *Partitiviridae*. Many partitiviruses are characterized by slow rates of mutation accumulation and genetic homogeneity within species (Jiang et al., 2015; Thapa et al., 2016; Petrzik, 2019), which was confirmed in this study for VCV isolates. Although the nucleotide diversity of VCV was generally very low (π = 0.0176–0.0359), Φst showed the differentiation of the VCV population from the wild grapevine from the Black Sea region from the isolates available in GenBank. This finding provides additional confirmation of the genetic isolation of the grapevine from the Black Sea region from other populations of *V. vinifera*.

To date, VCV has been identified in Japan, China, Russia, and France (Nabeshima and Abe, 2021; Fan et al., 2022; Shvets et al., 2022a; Candresse et al., 2024). VCV was first discovered in 2021 in Japan on the wild grapevine *V. coignetiae* (Nabeshima and Abe, 2021). Previous studies in ampelographic collections and commercial vineyards in southern Russia identified eight VCV isolates out of the 209 samples analyzed, representing 0%–6% of the different populations (**Figure 14**). It is interesting that five of them were found in various interspecies hybrids of *Vitis*, and one was found in an indigenous variety of the Crimean Peninsula. In China, VCV infected 12 of 470 analyzed cultivated grapevines (2.6%) that were interspecies hybrids of *Vitis* and *V. vinifera* ssp. *vinifera* (Fan et al., 2022). In France, VCV was also identified in interspecies hybrids (Candresse et al., 2024). The same researchers found VCV reads in the transcriptomes of *V. acerifolia, V. quinquangularis, V. romanetii, V. cinerea, V. davidii, V. amurensis*, and *V. vinifera* ssp. *sylvestris*.

VCV infection appears to be uncommon in most cultivars of *V. vinifera* ssp. *vinifera*; it is associated with other species and subspecies of *Vitis*, including *V. vinifera* ssp. *sylvestris* from the Black Sea region. Considering that plant partitiviruses are known to use only a vertical route of transmission (Vainio et al., 2018), VCV can be assumed to have an ancient history of interaction with different species of the genus *Vitis*.

The symptoms associated with the presence of VCV in cultivated or wild grapevine have not yet been identified (Candresse et al., 2024). Plant dsRNA viruses of the family *Partitiviridae*, which includes VCV, are characterized by persistent infections that do not have a clear negative effect on the host (Nibert et al., 2014; Vainio et al., 2018). Moreover, pepper cryptic virus 1 (*Deltapartitivirus unocapsici*), which also belongs to the family *Partitiviridae* and is found in most jalapeño pepper plants, is known to repel aphids and thus protect plants from transmitters of acute viruses (Safari et al., 2019). Therefore, further studies will help shed light on the role of VCV in the grapevine holobiont.

#### 4.3.2 Grapevine virus T

The distribution of GVT in wild grapevine (48%–50%) was greater than that in cultivated grapevine from the nearest viticultural region (8%–42%) (**Figure 14**). The GVT population from wild grapevine from the Black Sea region was characterized by relatively high genetic diversity (π = 0.153), which exceeded the diversity of isolates from cultivated grapevine of southern Russia (π = 0.118). A number of studies in commercial vineyards have reported high genetic diversity of GVT, even in a small area (Glasa et al., 2018; Zarghani et al., 2018).

Currently, genetic variants of GVT are divided into eight phylogroups (Zarghani et al., 2018; Demian et al., 2021). The isolates obtained in this study belonged to three of them, with some isolates clustering separately. On the one hand, the formation of unique genetic variants may indicate the long-term existence of the GVT population in the nature reserves from the Black Sea region. On the other hand, GVT was described for the first time relatively recently, in 2017 (Jo et al., 2017), and to date, GenBank has a limited number of complete genomes, which is insufficient to suggest that the detected variants are specific to the grapevine from the Black Sea region.

The vectors of GVT are unknown (Fuchs, 2020). Previous studies have noted the presence of GVT on cultivated grapevine in many European countries: Italy (Jo et al., 2017), Czech Republic and Slovakia (Glasa et al., 2018), France (Zarghani et al., 2018), Croatia (Vončina and Almeida, 2018), Germany (Ruiz-García et al., 2018), and Hungary (Demian et al., 2021). The fixation index calculated in this study indicates frequent genetic exchange between GVT populations from Europe and the Black Sea region (Φst = 0.055) and less intense exchange between populations from Europe and Russia (Φst = 0.155). The Φst value for populations from Russia and the Black Sea region was not statistically significant, but their close genetic relationships were evident from phylogenetic analysis.

The high genetic diversity and wide distribution of GVT in the samples suggest that this virus has long coexisted with *V. vinifera* ssp. *sylvestris* in the Black Sea region, and its distribution is hardly associated with human economic activity.

#### 4.3.3 Grapevine rupestris stem pitting–associated virus

Another widely represented virus in the wild grapevine from the Black Sea region was GRSPaV. This is one of the most widespread grapevine viruses in the world (Meng and Rowhani, 2017) and, in particular, in ampelographic collections and commercial vineyards in southern Russia (89%–98%) (**Figure 14**). In wild grapevine from the Black Sea region, GRSPaV was less widespread—70% in the population from the Utrish Nature Reserve and 28% from the Crimean Nature Reserve. GRSPaV was previously identified in wild grapevine. In Tuscany (Italy), GRSPaV infection was noted in 30% of *V. vinifera* ssp. *silvestris* plants (Sabella et al., 2018), in Tunisia—51% of plants (Selmi et al., 2020), and in Sicily (Italy)—12% (Pacifico et al., 2016).

An analysis of the GRSPaV population from wild grapevine from the Black Sea region revealed high genetic diversity (π = 0.163) and that the isolates belonged to four of the six described phylogenetic groups. These results are consistent with previous studies that noted the absence of geographic clustering of GRSPaV genetic variants (Alabi et al., 2010; Hily et al., 2018). In particular, GRSPaV isolates of different phylogenetic groups were found in the wild grapevine of Sicily (Pacifico et al., 2016). Moreover, the relationships between the phylogroups of the detected GRSPaV isolates from the Black Sea region and the genetic clusters of the host plants were not established.

The spread of different genetic variants of GRSPaV around the world is believed to be linked to human activity, particularly transmission through grafting (Meng and Rowhani, 2017). However, this does not explain the high genetic diversity of GRSPaV in wild grapevine, which may indicate the presence of another route of transmission. GRSPaV could have been transferred to wild grapevine from the Black Sea region from vineyards in southern Russia, where the viticulture industry is well developed. We have previously shown that GRSPaV isolates from all six phylogroups are present in these regions (Belkina et al., 2023).

#### 4.3.4 Grapevine pinot gris virus

The fourth most represented virus in this study was GPGV, which under certain conditions leads to the development of grapevine leaf mottling and deformation (GLMD) (Saldarelli et al., 2017).

GPGV was more widespread in cultivated grapevine from southern Russia (62%–96%) than in wild grapevine from the Black Sea region (22%–67%) (**Figure 14**). At the same time, GPGV was detected in 67% of the wild grapevines from the Utrish Nature Reserve, whereas in cultivated grapevines from the same region its prevalence was 90%–96%. On the Crimean Peninsula, the prevalence of GPGV in wild and cultivated grapevine populations was 22% and 67%, respectively. Thus, in the wild grapevine of both nature reserves from the Black Sea region, the prevalence of the virus is lower than that in the grapevine of the nearby studied vineyards.

The negative values of the Tajima statistic in all the GPGV populations analyzed in this study may indicate purifying selection, which maintains a relatively low level of genetic diversity. This is confirmed by the low values of the π index that for the set of global GPGV isolates was 0.028, which is consistent with the results of previous studies (Tarquini et al., 2019).

The results of the present study confirm the suggestion of Hily et al. about the Asian origin of GPGV (Hily et al., 2020). This is supported by the increased genetic diversity of GPGV isolates from Asia (π = 0.066) compared with other populations. It is assumed that GPGV entered Europe in the 20th century (Hily et al., 2020). The low Φst value (0.067) between the European and Russian populations established in this study indicates frequent genetic exchange; thus, it is likely that GPGV migrated from Europe to Russia along with infected planting material. Territorial proximity could contribute to intensive genetic exchange between GPGV populations from Russia and the Black Sea region, which was confirmed by the low Φst value (0.036) and the results of phylogenetic analysis. Presumably, the spread of GPGV was directed from commercial vineyards in Russia to wild grapevine from the Black Sea region but not vice versa, since the analyzed GPGV isolates from the Black Sea region were characterized by relatively low genetic diversity (π = 0.016).

The vast majority of Russian GPGV isolates do not have polymorphism at the end of the ORF encoding the MP, which leads to the formation of an early stop codon (Shvets and Vinogradova, 2022). This polymorphism affects the increase in GPGV titer and the manifestation of GLMD symptoms, which has been proven experimentally (Saldarelli et al., 2015; Bertazzon et al., 2017; Tarquini et al., 2021). All the GPGV isolates obtained in this study also did not contain the truncated MP. Thus, the wild grapevine from the Black Sea region is a potential reservoir of GPGV infection, but the strains circulating in the population are likely to be associated with asymptomatic infection.

For intensive genetic exchange to occur, a vector is necessary. GPGV is known to be transmitted through grafting (Saldarelli et al., 2015) and by the mite *Colomerus vitis*, which is a pest of grapevine (Malagnini et al., 2016). Moreover, according to the latest data, GPGV is capable of infecting not only grapevine but also herbaceous plants (Gualandri et al., 2017), as well as plants of the genera *Ailanthus, Asclepias, Crataegus, Fraxinus, Rosa, Rubus*, and *Sambucus* (Demian et al., 2022). Therefore, an unknown vector must exist. This allows us to assume that the invasion of GPGV into the protected areas of the Black Sea region may have been carried out not from the grapevines of neighboring vineyards, but from other plant species. Future research should help shed light on this issue.

### 4.4 Wild grapevine from the Black Sea region: a source of novel viruses

#### 4.4.1 Abrau grapevine-associated virus

AGaV was discovered in this study, and the viruses closest in phylogeny form a clade with high bootstrap support that clusters close to several families from the order *Hepelivirales*. The order *Hepelivirales* includes (+) ssRNA viruses whose hosts belong to several kingdoms. AGaV clusters most closely with beny-like viruses found in insects (Huang et al., 2021) and fungi (Picarelli et al., 2019), although most of them have been submitted to GenBank without scientific publication. Moreover, the genome organization of these viruses differs from AGaV, which is predicted to possess three ORFs. Such genome organization with three ORFs is characteristic of the family *Hepeviridae*, which includes monopartite viruses of vertebrates (Purdy et al., 2022), as well as unclassified beny-like viruses found in plant transcriptomes (Solovyev and Morozov, 2022). On the other hand, the amino acid sequence of the replication-associated polyprotein and the conserved protein domains of AGaV bring it closer proximity to plant viruses of the family *Benyviridae*. However, benyviruses have a multipartite genome and a single ORF on RNA1 (Gilmer and Ratti, 2017). In summary, we can assume that AGaV has a monopartite genome, similar to viruses of the family *Hepeviridae*, and its ORF2 appears to encode the coat protein. Further studies are necessary to determine the true host of AGaV.

Taking into account the discovery of novel viruses with different genome organizations that cluster separately from viruses from the approved ICTV families, the taxonomy of the order *Hepelivirales* needs to be revised.

#### 4.4.2 Taurida grapevine-associated virus

The second novel virus discovered in this study, TGaV, belongs to the order *Picornavirales*, which includes (+) ssRNA viruses with different variants of genome organization (Le Gall et al., 2008). Some families in this order (*Dicistroviridae* and *Marnaviridae*) have a bicistronic genome, similar to TGaV. However, in these families, ORF1 encodes the replication complex and ORF2 encodes the structural polyprotein, whereas in TGaV the structural polyprotein is located at the 5’ end. This type of genome organization has been described for a number of unclassified picorna-like viruses, such as cherry virus Trakiya, aphis glycines virus 1, Tetranychus urticae-associated picorna-like virus 1, rice curl dwarf-associated virus, etc (Milusheva et al., 2019; Koloniuk et al., 2020; Yasmin et al., 2020; Zhang et al., 2021). These viruses form a clade (which includes TGaV) with high bootstrap support in dendrograms based on structural and nonstructural proteins; possibly, they are representatives of a novel family in the order *Picornavirales* (Yasmin et al., 2020). Viruses of this group were discovered in the analysis of arthropods, fungi, and plants, including symptomatic rice and cherry plants (Zhang et al., 2021; Milusheva and James, 2022). Therefore, further studies will be required to accurately determine the host of TGaV.

## 5 Conclusions

The wild grapevine populations of the Crimean and Utrish nature reserves from the Black Sea region analyzed in this study belonged to *V. vinifera* ssp. *sylvestris* and were genetically close to *V. vinifera* ssp. *sylvestris* from southeastern Europe. The wild grapevine population from Abkhazia was related to both native Abkhazian and Georgian varieties and the Caucasian *V. vinifera* ssp. *sylvestris*.

The viral communities of the studied populations of wild grapevine from the Black Sea region differed significantly from both the viromes of cultivated grapevine in southern Russia and the viromes of wild grapevine in other countries. Despite the genetic differentiation between the wild grapevine populations from the Black Sea region and the cultivated varieties, most of the identified viral isolates were similar to available world accessions, probably due to the presence of vectors for some viruses.

In the wild grapevine virome from the Black Sea region, VCV, GVT, GRSPaV, and GPGV were predominant. Apparently, GPGV is not an indigenous pathogen for the wild grapevine from the Black Sea region, and its spread may be associated with the globalization of viticulture and the presence of vectors other than the mite *Colomerus vitis*. In contrast, two related viruses, GVT and GRSPaV, for which vectors are currently unknown, have probably been maintained in the wild grapevine population from the Black Sea region for a long period of time. VCV, which, unlike GPGV, GRSPaV, and GVT, is not common in cultivated grapevine, was dominant in the wild grapevine from the Black Sea region. Since VCV is transmitted vertically and was found on many *Vitis* species and interspecies hybrids, this suggests that during grapevine domestication, a population in which VCV was absent became widespread.

In addition to new data on already known viruses, we discovered two novel viruses in the wild grapevine from the Black Sea region: AGaV in the order *Hepelivirales* and TGaV in the order *Picornavirales*.

Research on the genetics of *V. vinifera* ssp. *sylvestris* and its viruses, both in the Black Sea region and in other centers of grapevine domestication, will help to develop conceptions about natural populations of viruses that arose during coevolution with grapevine, their phylogeography, and their role in the ecosystem. All of these findings will contribute to the further study of grapevine domestication.

## Data Availability

The datasets presented in this study can be found in online repositories. The names of the repository/repositories and accession number(s) can be found below: https://www.ncbi.nlm.nih.gov/genbank/, PRJNA1148509.
