# Supplementary material for: In-depth population genetic study of Vitis vinifera ssp. sylvestris from the Black Sea region and its virome
Source: Front Plant Sci. 2025 Mar 25;16:1536862. doi: 10.3389/fpls.2025.1536862 (PMC11975898; doi:10.3389/fpls.2025.1536862)
Supplement: Supplementary file 1 [file DataSheet1.pdf]

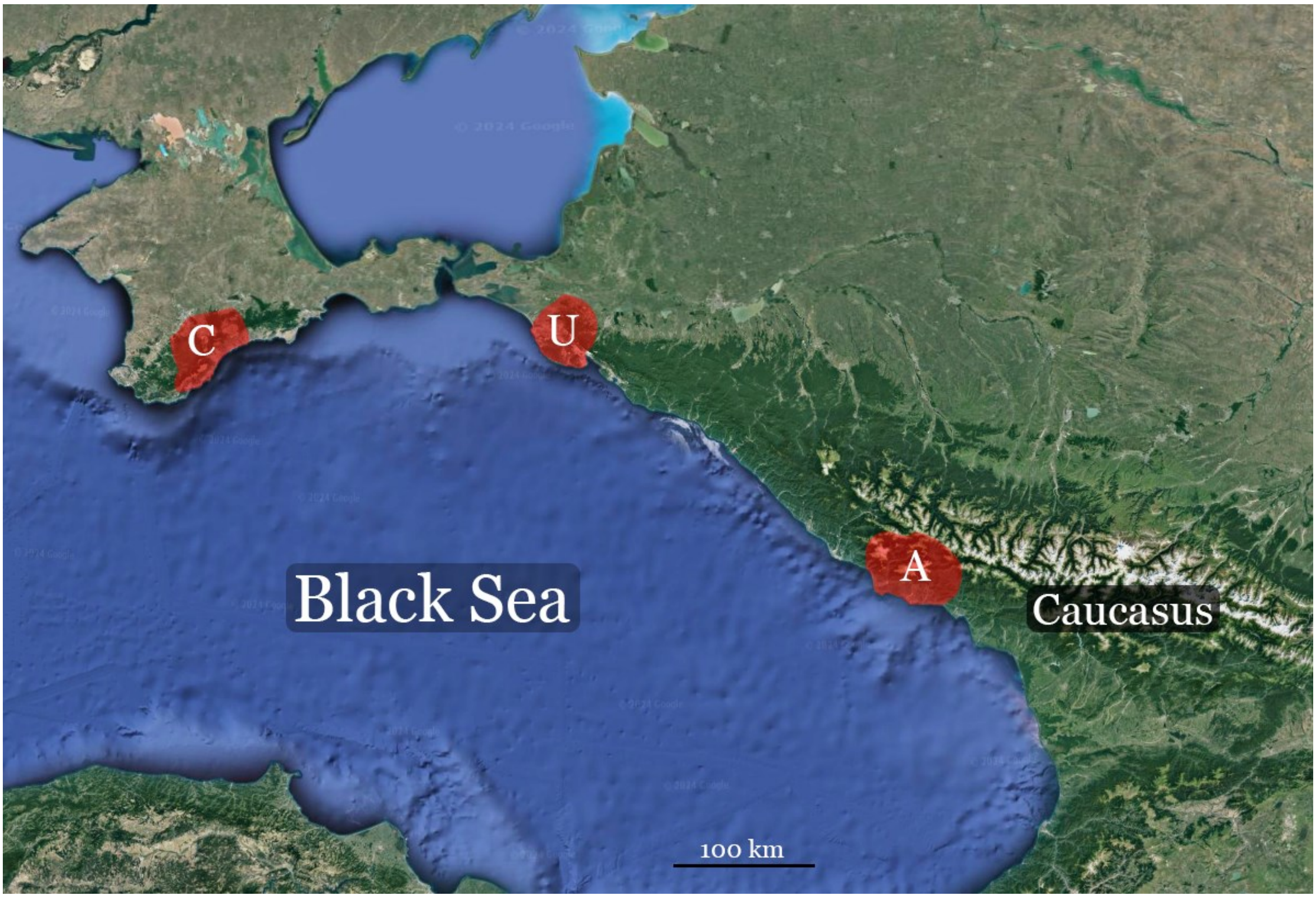

Supplementary Figure 1. Wild grapevine sampling areas. C – Crimean Nature Reserve; U – Utrish State Nature Reserve; A – the mountain forests of Abkhazia.

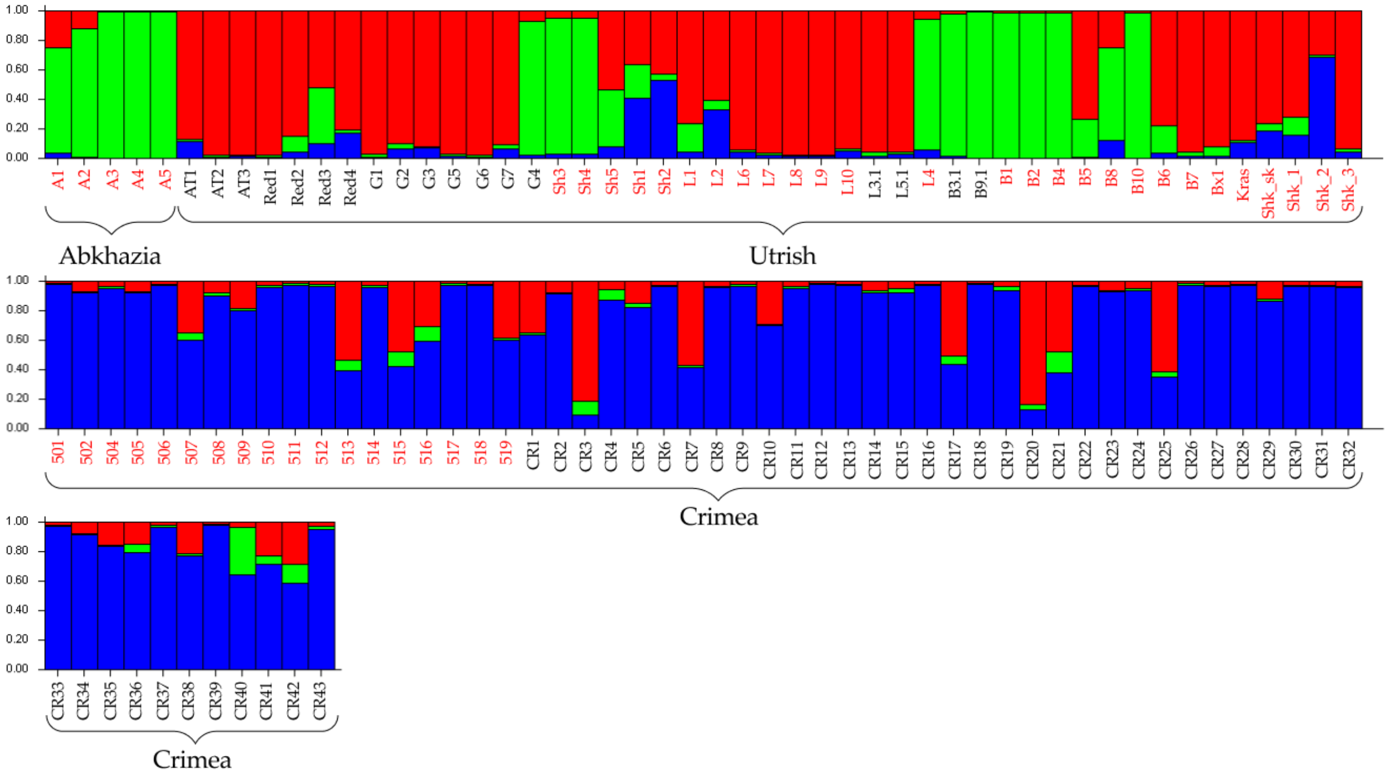

Supplementary Figure 2. Population structure analysis of wild grapevine of the Black Sea region using STRUCTURE software at K = 3.

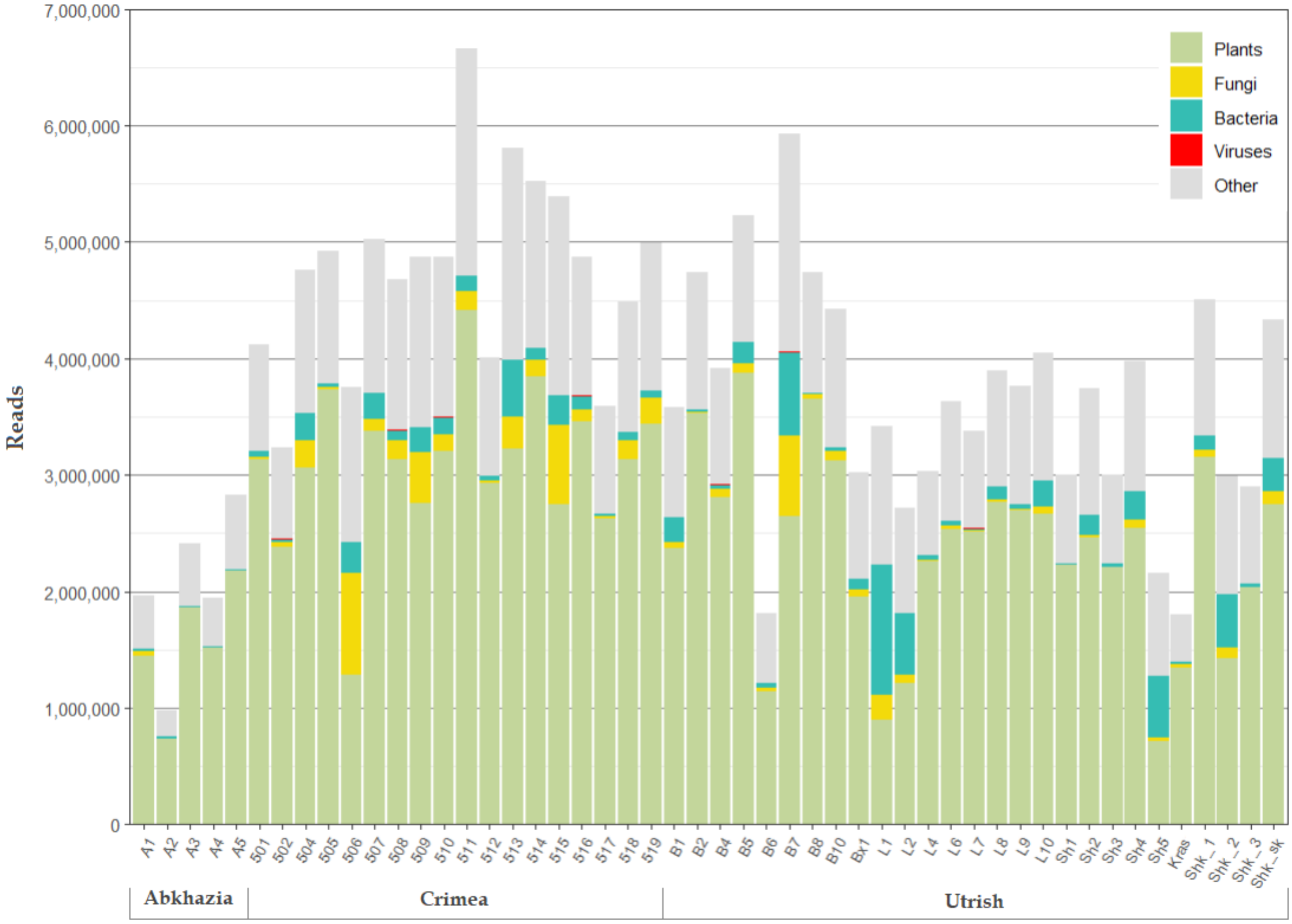

Supplementary Figure 3. Number of the preprocessed reads assigned by Kraken2 to different groups of organisms for each library. Columns represent samples from three areas of Black Sea region.

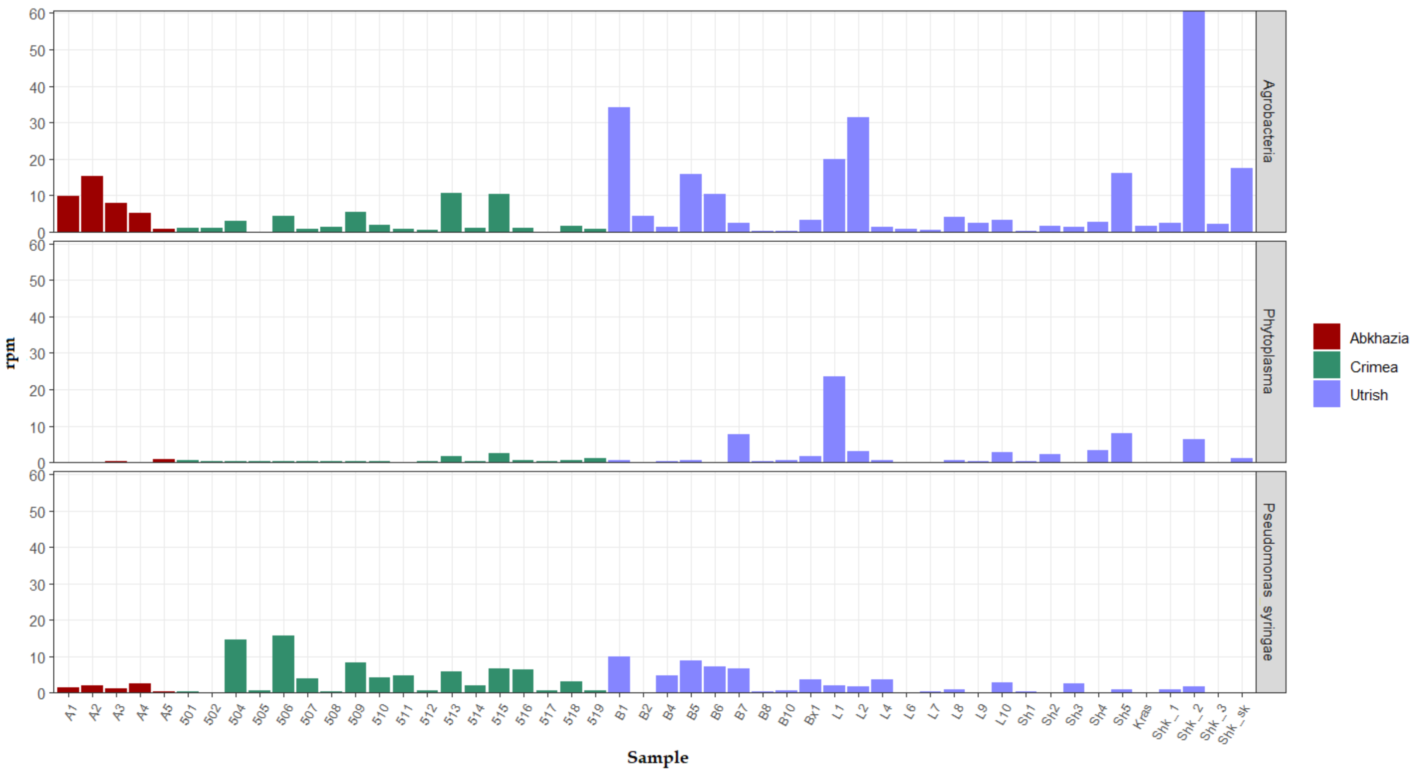

Supplementary Figure 4. Proportion of the preprocessed reads assigned by Kraken2 to the plant pathogenic bacteria in reads per million (rpm) for each library. The species *Agrobacterium tumefaciens, Allorhizobium ampelinum,* and *Agrobacterium vitis are* grouped under the heading Agrobacteria.

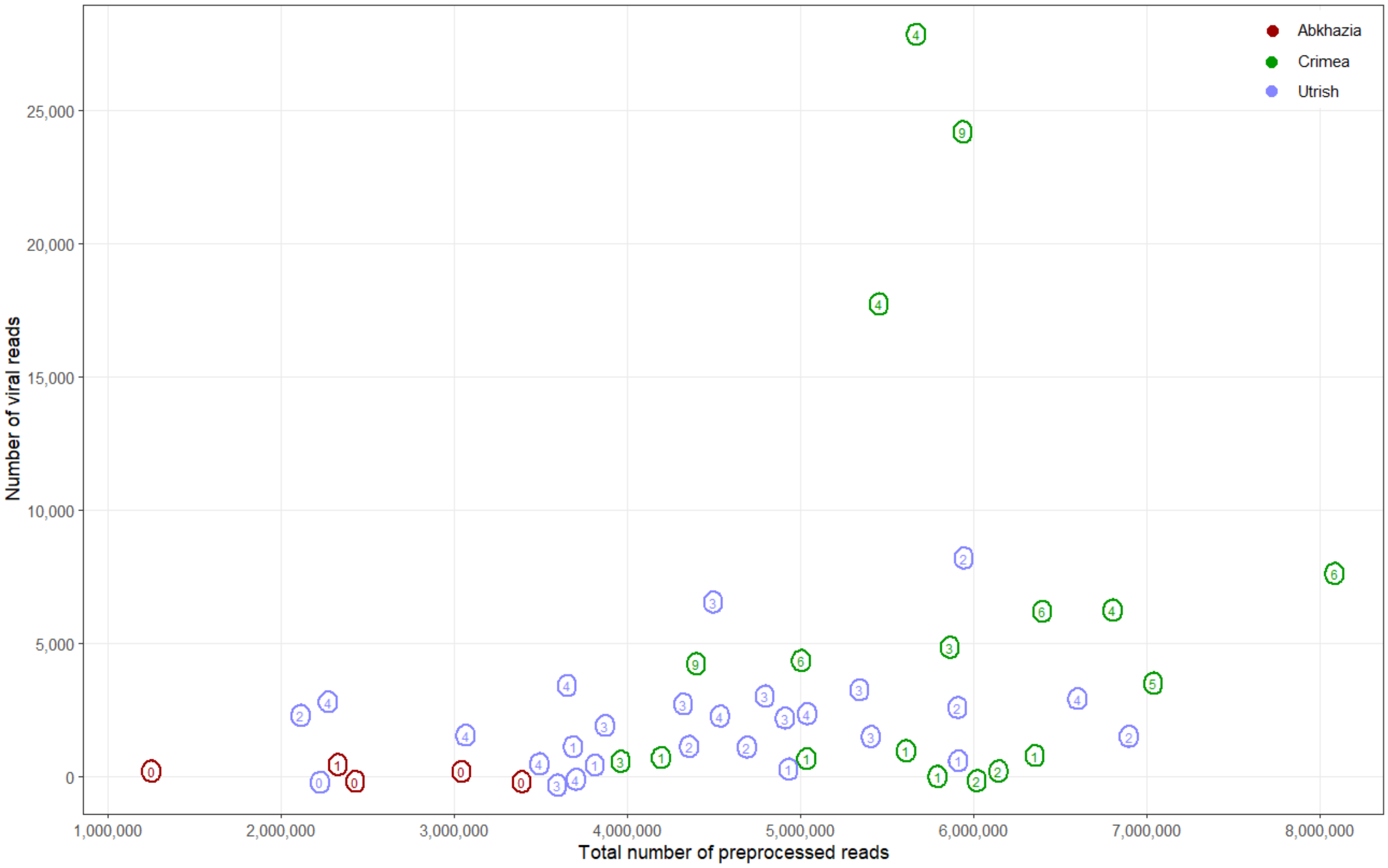

Supplementary Figure 5. Relations between the number of reads mapped to viral reference genomes and total number of preprocessed reads. Each dot represents one library (one plant). The digit inside the dot shows the number of viruses detected in that plant.

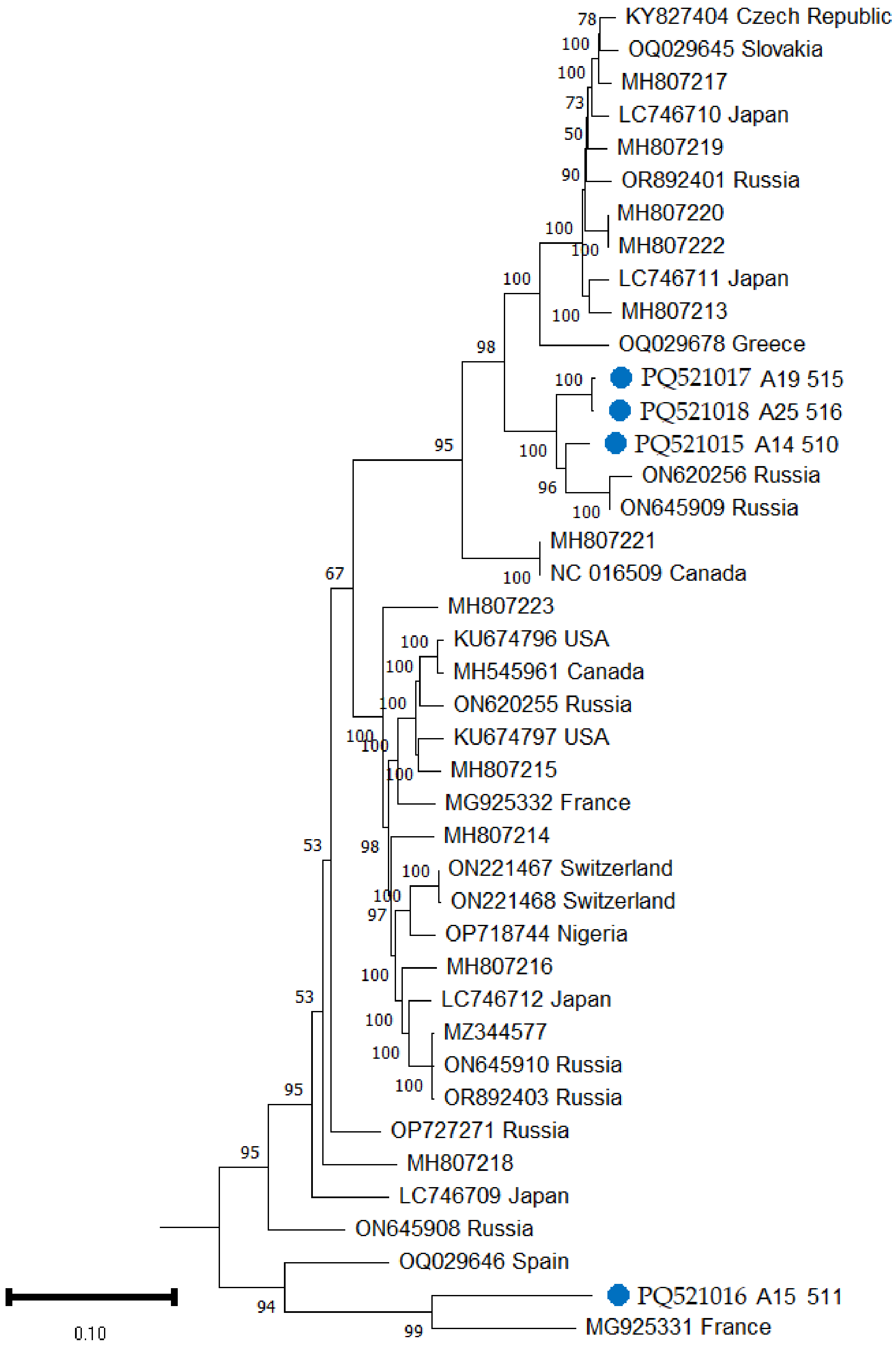

Supplementary Figure 6. Phylogenetic analysis based on genome sequences of grapevine leafroll-associated virus 1 (GLRaV-1) isolates obtained in this study (blue dots), and world isolates. Tree was constructed in MEGA11 using the maximum likelihood method and GTR model with 1000 bootstrap replicates.

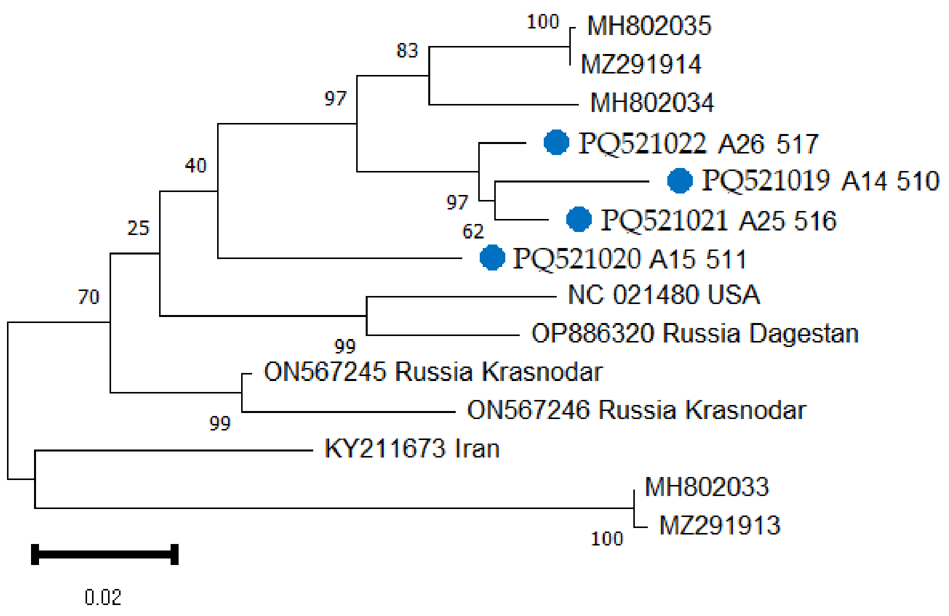

Supplementary Figure 7. Phylogenetic analysis based on complete genome sequences of grapevine satellite virus (GV-Sat) isolates obtained in this study (blue dots), and world isolates. Tree was constructed in MEGA11 using the maximum likelihood method and T92 model with 1000 bootstrap replicates.

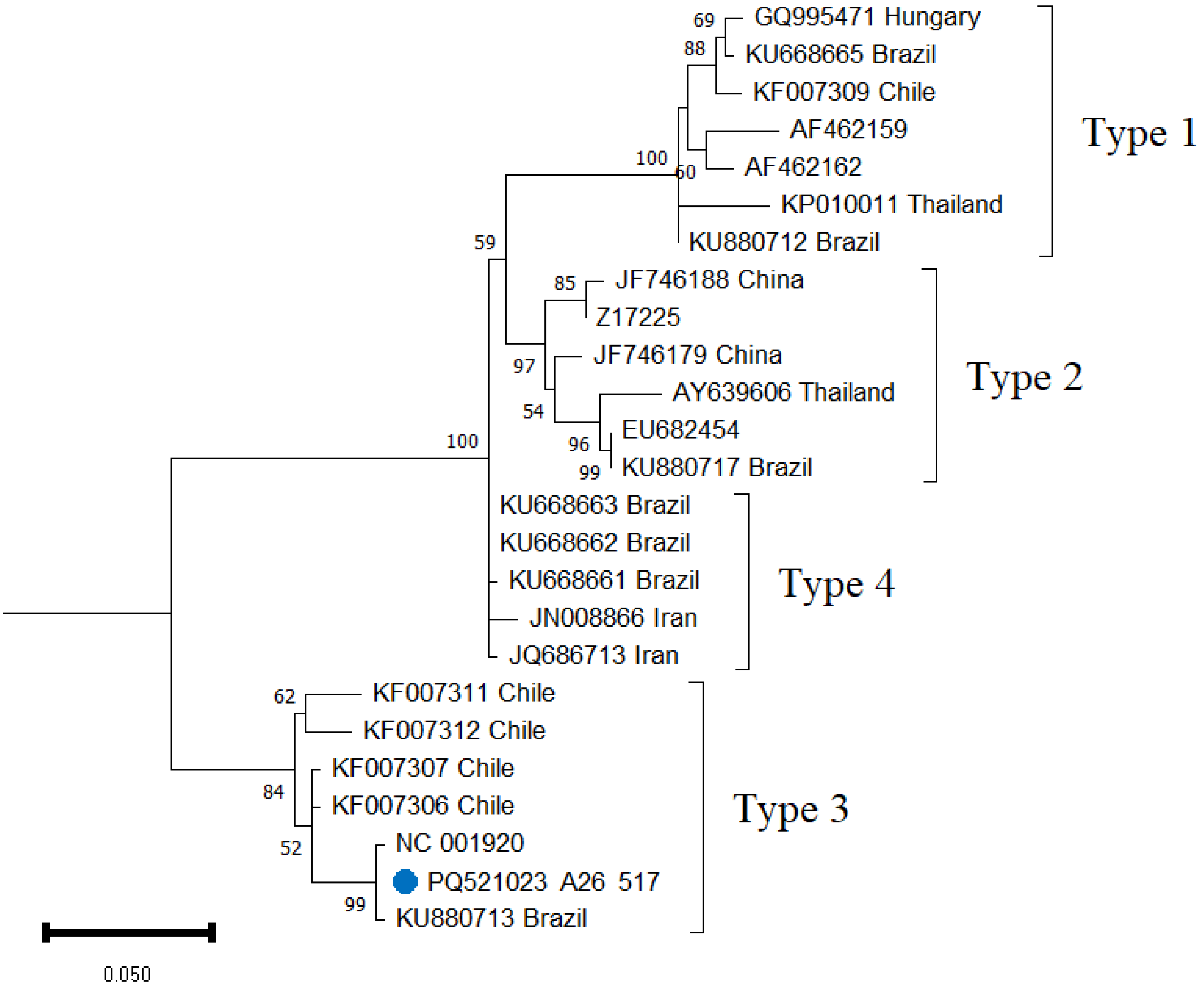

Supplementary Figure 8. Phylogenetic analysis based on complete genome sequences of grapevine yellow speckle viroid 1 (GYSVd-1) isolates obtained in this study (blue dot), and representative phylogroup members. Tree was constructed in MEGA11 using the maximum likelihood method and T92 model with 1000 bootstrap replicates.

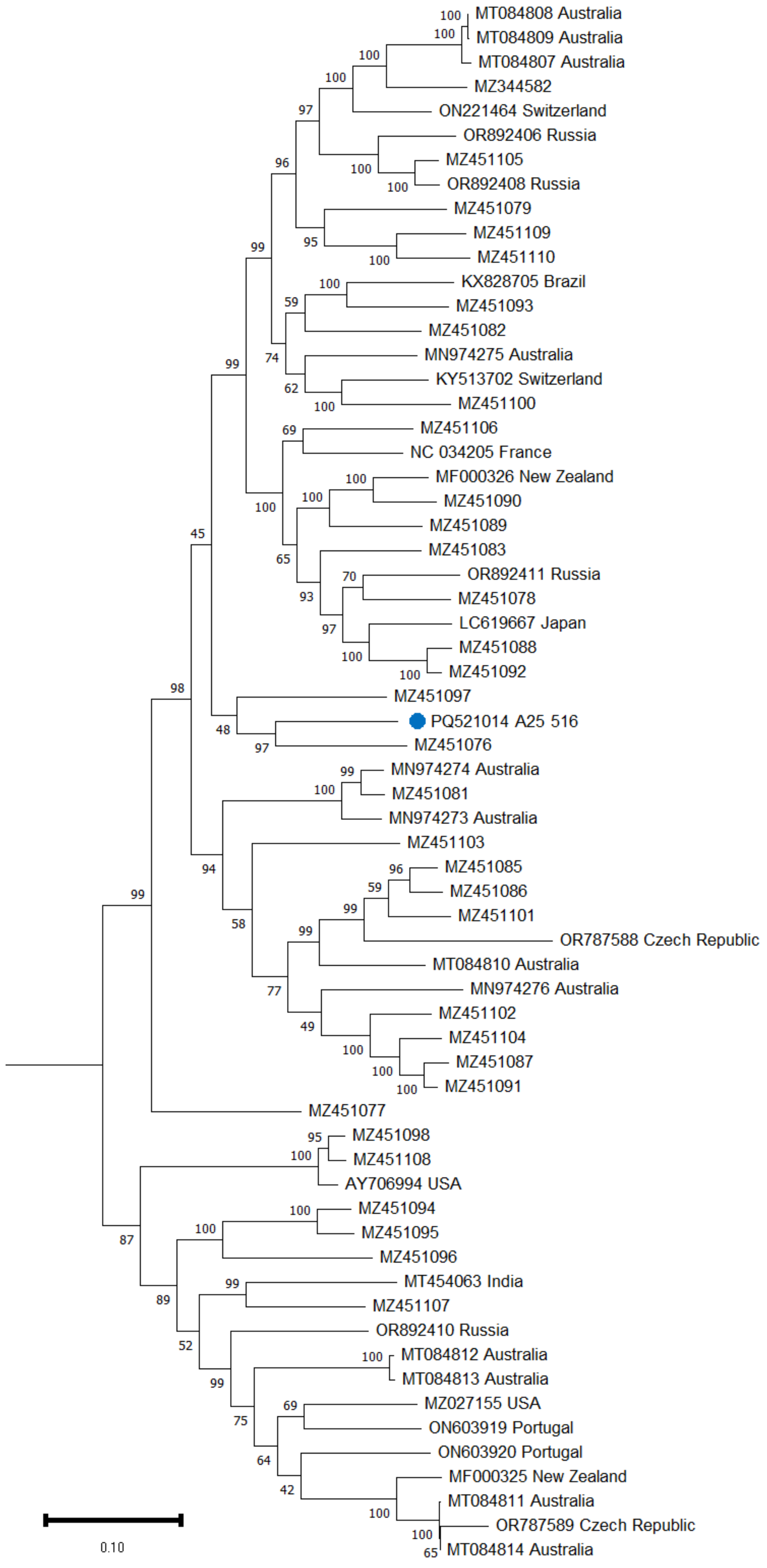

Supplementary Figure 9. Phylogenetic analysis based on complete genome sequences of grapevine rupestris vein feathering virus (GRVFV) isolates obtained in this study (blue dot), and world isolates. Tree was constructed in MEGA11 using the maximum likelihood method and GTR model with 1000 bootstrap replicates.

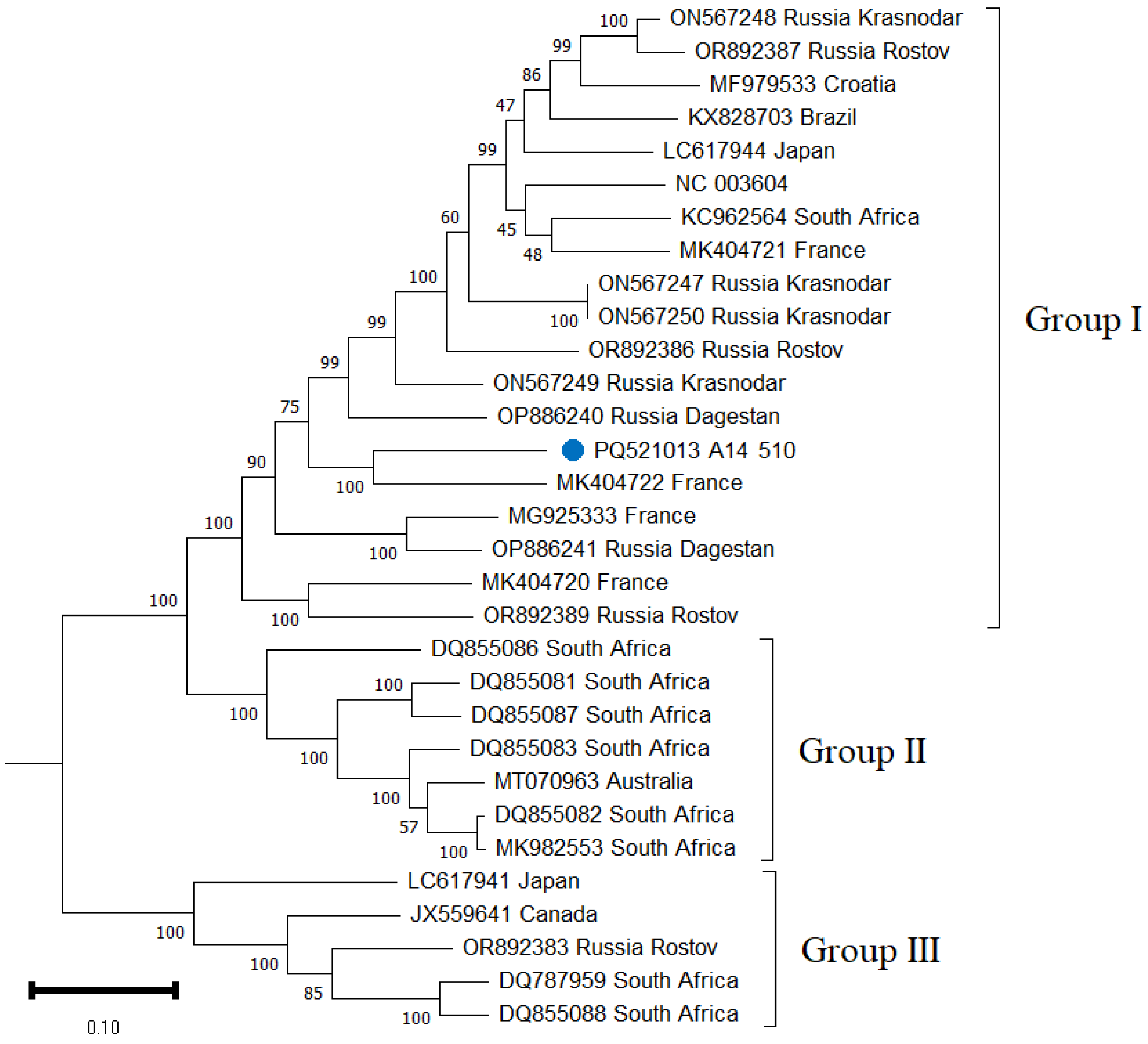

Supplementary Figure 10. Phylogenetic analysis based on complete genome sequences of grapevine virus A (GVA) isolates obtained in this study (blue dot), Russian isolates, and representative phylogroup members. Tree was constructed in MEGA11 using the maximum likelihood method and GTR model with 1000 bootstrap replicates.

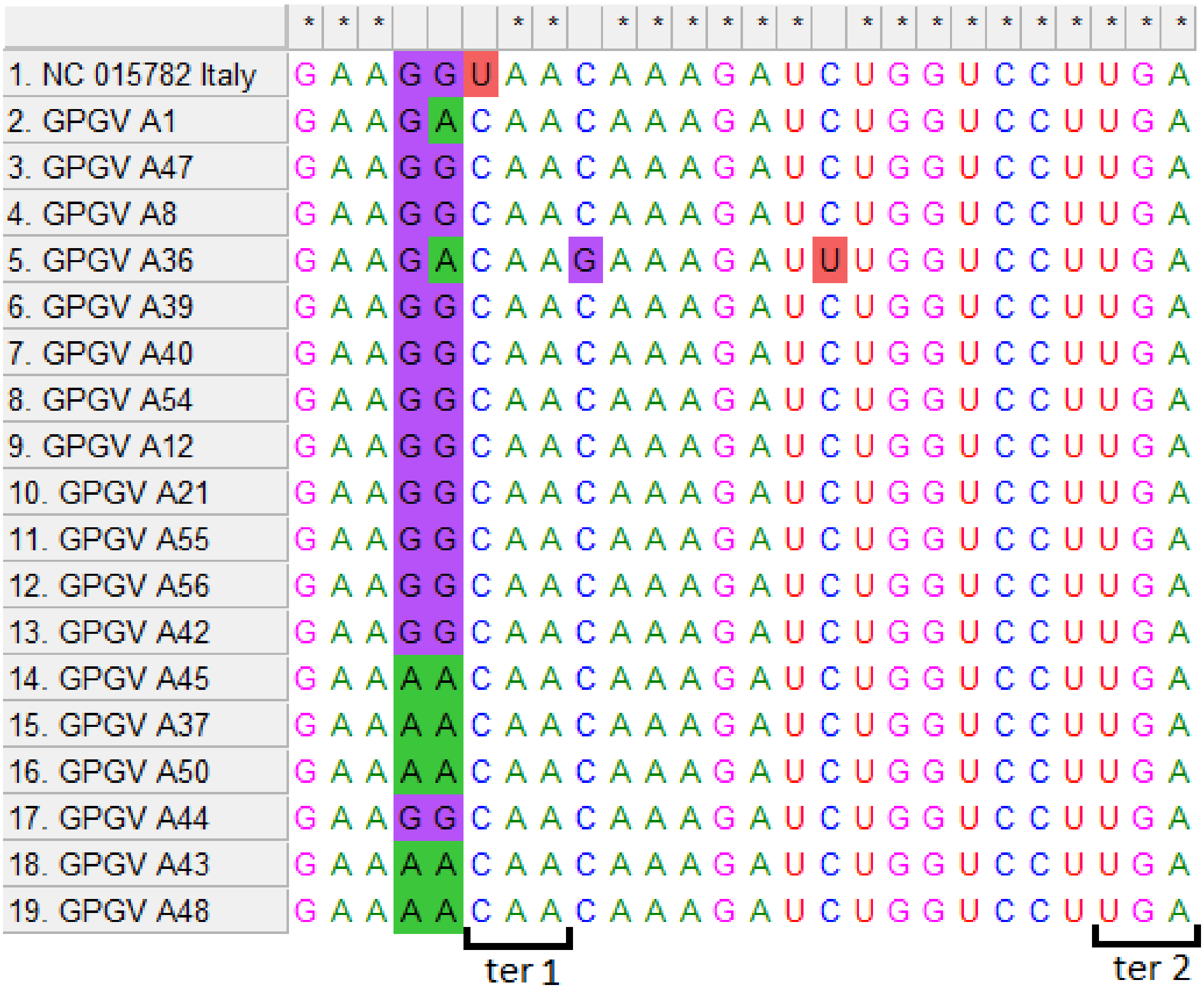

Supplementary Figure 11. Multiple alignment of the terminal fragment of the MP-encoding ORF of grapevine pinot gris virus (GPGV) isolates from wild grapevines in the Black Sea region and the reference GPGV genome. An early stop codon associated with grapevine leaf mottling and deformation is indicated as ter1. A stop codon prevalent in asymptomatic strains is indicated as ter2.
